# Mild synthesis of isoxazoline derivatives via an efficient [4 + 1] annulation reaction of transient nitrosoalkenes and sulfur ylides

**DOI:** 10.1038/s41598-021-81370-w

**Published:** 2021-01-22

**Authors:** Ting-Bi Hua, Cheng-Xiong Liu, Wei-Min Hu, Long Wang, Qing-Qing Yang

**Affiliations:** 1 Key Laboratory of Inorganic Nonmetallic Crystalline and Energy Conversion Materials, College of Materials and Chemical Engineering, China Three Gorges University, Yichang, 443002 Hubei China; 2 Hubei Key Laboratory of Natural Products Research and Development, China Three Gorges University, 8 Daxue Road, Yichang, 443002 Hubei China

**Keywords:** Chemistry, Organic chemistry, Synthetic chemistry methodology

## Abstract

An efficient [4 + 1] annulation between *α*-bromooximes and sulfur ylides via in situ generation of nitrosoalkenes under mild basic reaction conditions has been developed, providing an expeditious and scalable approach to synthesize biologically interesting isoxazoline derivatives with good to excellent yields.

## Introduction

**Figure 1:**
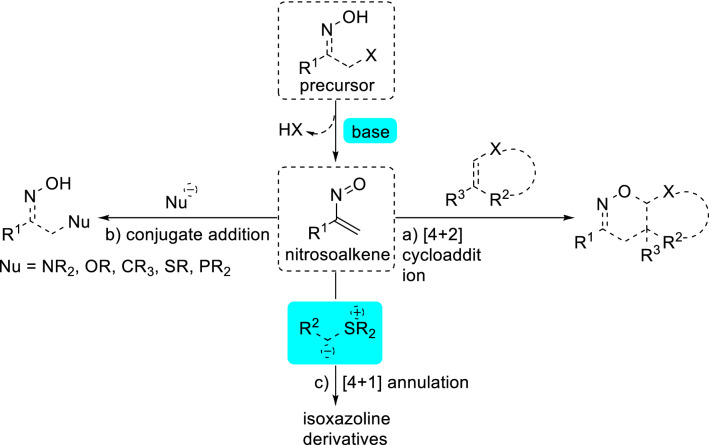
Synthetic transformation of transient nitrosoalkenes from α-halooximes: (**a**) [4 + 2] cycloaddition with electron-rich heterocycles or nucleophilic olefins; (**b**) conjugate addition reactions with various nucleophiles; (**c**) [4 + 1] annulation with sulfur ylides.

Nitrosoalkenes are considered as highly reactive and versatile synthetic intermediates in organic synthesis^1–5^. This is mainly due to easy access from available precursors: the base-mediated dehydrohalogenation of α-halooximes. As shown in Fig. 1, the most important synthetic application of this valuable intermediate is their use as an electron-deficient heterodiene to participate in [4 + 2] cycloaddition with electron-rich heterocycles or nucleophilic olefins (Fig. 1a)^6,7^ and as Michael-type acceptors in conjugate addition reactions (Fig. 1b)^8^, which are effective synthetic methodologies to 1,2-oxazines and open-chain oximes^9,10^.

However, despite the impressive advances that have been achieved, to the best of our knowledge, the formation of biologically interesting five-membered heterocycles^11^ from the reaction of nitrosoalkene intermediates is much less studied^12^. In addition, [4 + 1] annulation strategy represents a straightforward protocol for the construction of five-membered rings^13–15^. Sulfur ylides are vital C1 units that have been widely applied for the construction of five-membered rings via [4 + 1] annulation reactions^16–33^. For example, the groups of Tang and Xiao independently reported the [4 + 1] annulation of nitroolefines and stable sulfur ylides to give isoxazoline derivatives^17,18^. Based on these elegant results, we imagined that if nitrosoalkenes could be a good annulation partner with sulfur ylides, since it can be easily generated in situ via the dehydrohalogenation of α-halooximes. In this regard, Bolm’s group recently reported the first example of copper-catalyzed asymmetric formal [4 + 1] cyclization of in situ generated azoalkenes with stable sulfur ylides to synthesize chiral dihydropyrazoles^19,29^. Subsequently, Xiao and co-workers have described highly efficient indole and indoline synthesis that relies on [4 + 1] reactions of stable sulfur ylides and aza-*o*-quinodimethane intermediate generated in situ^20,21^. As a part of our interest in the reactions with sulfur ylide^14,25^ and developing efficient synthetic protcols towards the construction of biologically interesting heterocycles^34–41^, we envision that sulfur ylides would be the perfect C1 units to react with nitrosoalkene intermediates generated from α-bromooximes to afford isoxazoline derivatives (Fig. 1c), which are significant motifs existing in pharmaceutical and medicinal chemistry^42–50^.

## Results and discussion

We test our hypothesis with the easily prepared α-bromooxime **1a** and the phenylacyl-substituted sulfur ylide **2a** as model substrates. The initial experiment was carried out with potassium carbonate (1.2 equiv.) as base in CH_2_Cl_2_ at room temperature. Gratifyingly, the model reaction successfully took place and the desired product **3aa** was obtained in a good yield of 71% (Table 1, entry 1). Intrigued by this preliminary result, we examined a variety of reaction parameters to improve product yield and the related key results are illustrated in Table 1. Firstly, the effect of the solvent for this transformation was investigated, and it was shown that CHCl_3_ was identified as an ideal medium for the generation of **3aa** (Table 1, entries 1–5). Once an efficient solvent was found, crucial for the success of this reaction is the choice of a suitable base, which could promote the in situ generation of the nitrosoalkene intermediates. Therefore, a brief screening of the base was conducted (Table 1, entries 6–9). Fortunately, the yield was improved to 89% by use of sodium carbonate (Table 1, entry 6). Further adjusting the amount of the base and the substrate ratio increased the yield to 94% (Table 1, entries 10 and 11). Finally, we have tried to change the leaving group of the oxime but unfortunately, no satisfactory result was obtained (Table 1, entry 12).

**Table 1:** Optimization of the reaction conditions^a^.


Entry	X	Solvent	Base (equiv.)	Yield^b^ (%)
1	Br	CH_2_Cl_2_	K_2_CO_3_ (1.2)	71
2	Br	CHCl_3_	K_2_CO_3_ (1.2)	80
3	Br	Et_2_O	K_2_CO_3_ (1.2)	62
4	Br	DMSO	K_2_CO_3_ (1.2)	26
5	Br	toluene	K_2_CO_3_ (1.2)	37
6	Br	CHCl_3_	Na_2_CO_3_ (1.2)	89
7	Br	CHCl_3_	NaOH (1.2)	48
8	Br	CHCl_3_	*t*-BuOK (1.2)	38
9	Br	CHCl_3_	Et_3_N (1.2)	20
10	Br	CHCl_3_	Na_2_CO_3_ (1.3)	91
11^c^	Br	CHCl_3_	Na_2_CO_3_ (1.3)	94
12^c^	Cl	CHCl_3_	Na_2_CO_3_ (1.3)	83

^a^Reaction conditions: **1a** (0.40 mmol), **2a** (0.48 mmol), solvent (4 mL), base. ^b^Yield of isolated product. ^c^The ratio of **1a** or **1a**ʹ and **2a** is 1:1.3. DMSO: dimethyl sulfoxide.

**Figure 2:**
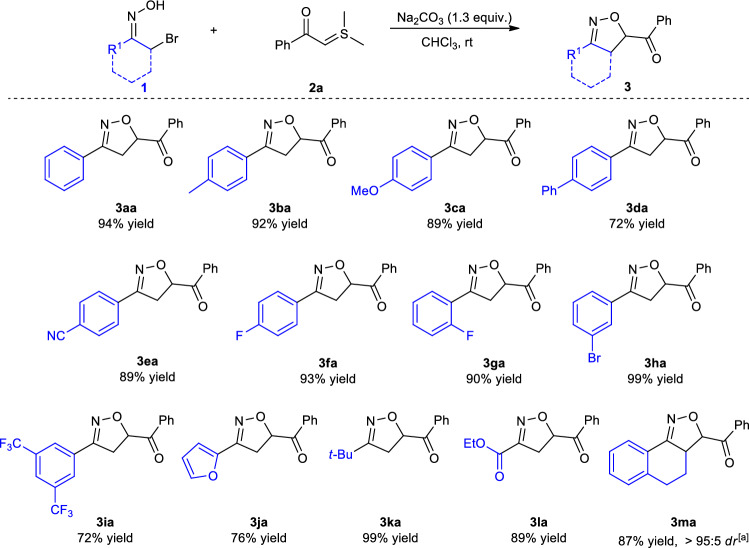
[4 + 1] annulation of various α-bromooximes **1** with sulfur ylide **2a.**

With the optimal reaction conditions established (Table 1, entry 11), we started to examine the generality of this [4 + 1] annulation reaction (Fig. 2). Firstly, we explored the tolerance of the functional substitution of substrates **1**. A broad range of *α*-bromooximes **1** underwent the annulation reaction to furnish products **3** in 72–99% yields. For instance, α-bromooximes substituted by either an electron-donating or -withdrawing group at the 4-position of benzene ring were well tolerated in this transformation, affording products with 72–94% yields (**3aa**-**3fa**). Substituents at the *ortho* or *meta* position on the benzene ring also react well (**3ga**, 90% yield and **3ha**, 99% yield). Significantly, α-bromooximes with two CF_3_ substituents on the benzene ring also gave a good yield of **3ia**. It is also worth noting that heteroaromatic.

*α*-bromooxime, such as furyl-substituted, was successfully applicable to the desired product **3ja**. Furthermore, the scope of *α*-bromooximes could be smoothly extended to alkyl- and ester-substituted *α*-bromooximes to provide the corresponding products **3ka** and **3la** in 99% and 89% yield, respectively. Additionally, introduction of tetrahydronaphthalene in the *α*-bromooximes could proceed efficiently in the reaction to afford the tricyclic product **3ma** in high yield and excellent diastereoselectivity.

**Figure 3:**
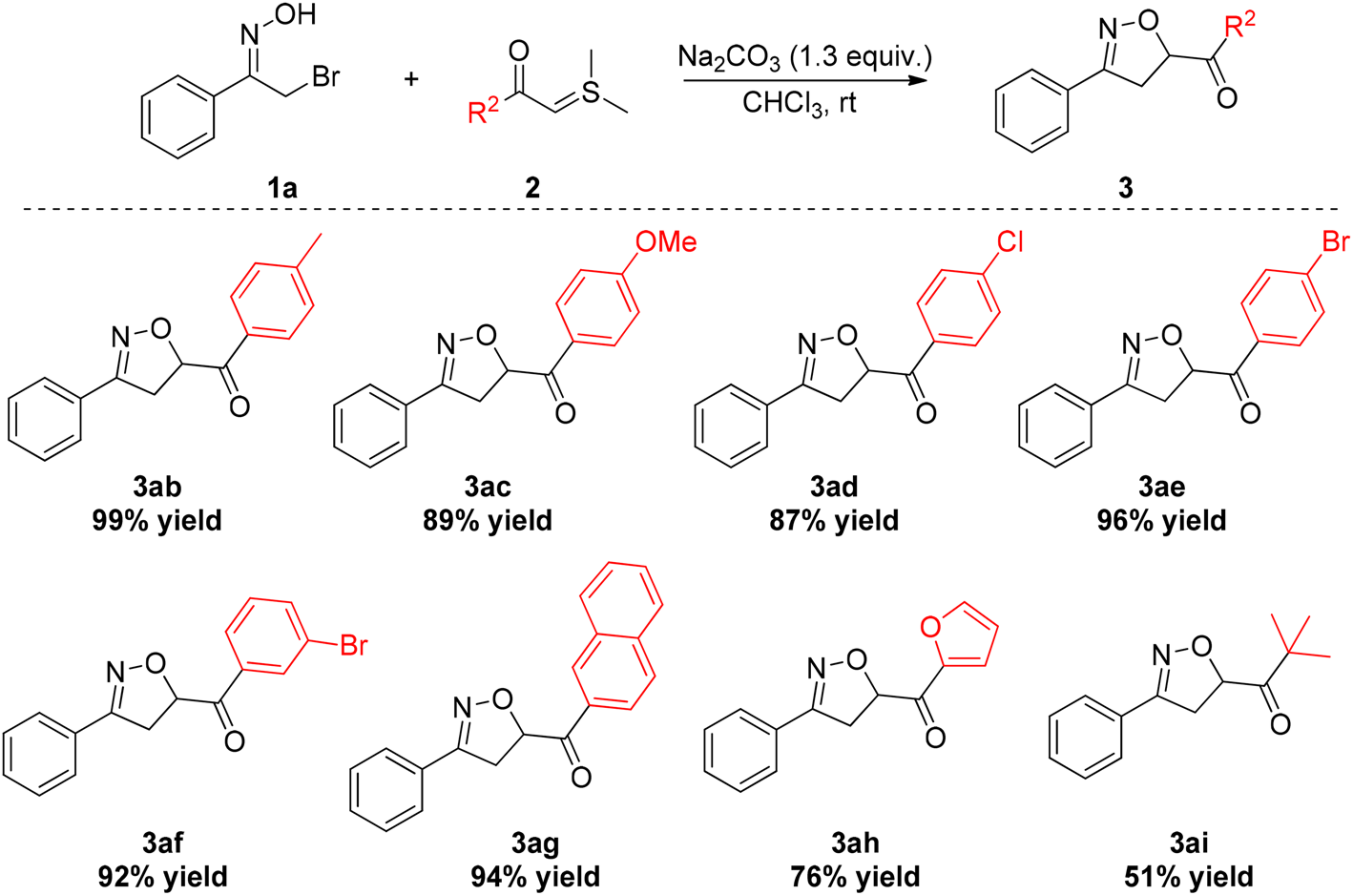
[4 + 1] annulation of α-bromooxime **1a** with various sulfur ylides **2.**

Of equal significance is the observation that structural modification of the sulfur ylide substrate is possible. As depicted in Fig. 3, substrates with electron-donating groups (4-Me, 4-OMe) and inductive electron-deficient groups (4-Cl, 4-Br, 3-Br) on the benzene ring were proven to react smoothly with **1a**, delivering the corresponding products **3ab**-**3af** in generally high to excellent yields. Moreover, naphthol-derived and heteroaromatic acyl sulfur ylides were also applicable as appropriate substrates for this transformation as products **3ag** and **3ah** were provided in 94% yield and 76% yield, respectively. In addition, the reaction with alkylacyl-substituted sulfur ylide **2j** proceeded successfully to afford the desired product in 51% yield.

**Figure 4:**
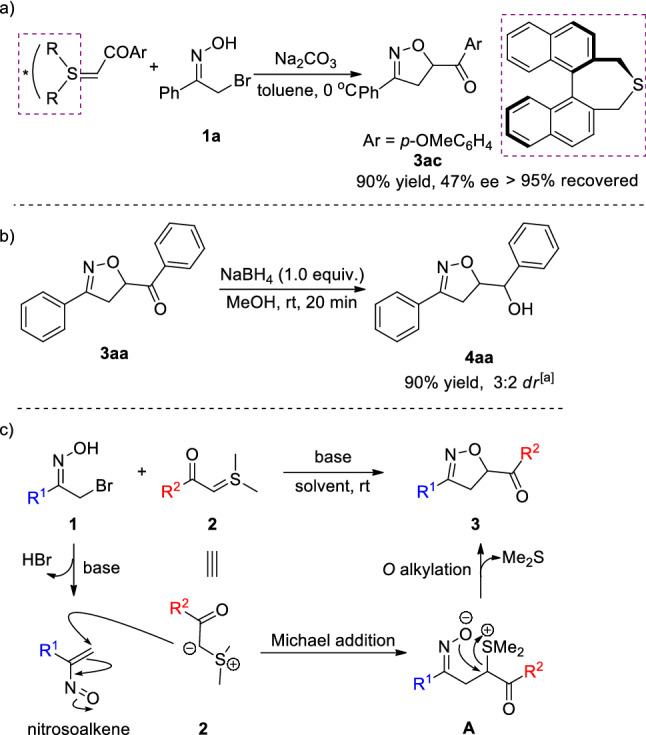
(**a**) Asymmetric reaction using (*R*)-BINOL derived chiral sulfur ylide; (**b**) Synthetic transformation of product **3aa**; ([**a**]: The diastereomeric ratio was determined via ^1^H NMR analysis of the crude reaction mixture.) (**c**) proposed mechanism for the [4 + 1] annulation reaction.

Based on previous works about enantioselective [4 + 1] annulation reactions of (*R*)-BINOL derived chiral sulfur ylide^13,16,21^, we applied this strategy to examine the asymmetric version in our process. To our delight, the enantioselective reaction indeed worked and product **3ac** was isolated with 47% ee, which could not be achieved with the corresponding precursor: (*R*)-BINOL derived chiral sulfonium salts due to the harsh reaction condition for the transformation from the related (*R*)-BINOL derived chiral sulfonium salts to sulfur ylide^15,21^. In an attempt to show the value of 2-isoxazolines as building blocks in synthesis, we tested a follow-up reduction reaction with Sodium borohydride in MeOH at room temperature. The desired product was obtained in 90% yield with 3:2 dr value after 20 min (Fig. 4a). A plausible mechanism for this [4 + 1] annulation reaction based on our experimental results and previous reports^5,19–21^ is illustrated in Fig. 4b. First, the nitrosoalkenes are generated in situ via a 1,4-elimination reaction under basic conditions. Then, a Michael addition occurs between the sulfur ylides **2** and the nitroalkenes, followed by an intramolecular *O* alkylation with loss of dimethylsulfide, delivering the final product **3**.

## Conclusion

In conclusion, we have reported an facile [4 + 1] annulation reaction between in situ formed nitrosoalkene intermediates and sulfur ylides via a Michael/intramolecular *O* alkylation sequence. The protocol provides a direct and efficient entry into valuable 2-isoxazoline derivatives in good to excellent yields. The reaction takes place under relatively mild and simple experimental conditions.

## Methods

General procedure for the synthesis of isoxazoline derivatives **3**: To a solution of sulfur ylide **2** (0.52 mmol) in trichloromethane (4.0 mL), Na_2_CO_3_ (0.52 mmol) was added and stirred at room temperature for 15 min. Then *α*-bromooxime **1** (0.40 mmol) was added at room temperature. The reaction was monitored via TLC (petroleum ether/ether = 10:3). Upon consumption of the starting materials, the reaction mixture was purified by flash chromatography on silica gel (petroleum ether/ether = 10:1) to give the desired product **3**.

## Supplementary Information

Supplementary Information.
