# Supplementary material for: Mild synthesis of isoxazoline derivatives via an efficient [4 + 1] annulation reaction of transient nitrosoalkenes and sulfur ylides
Source: Sci Rep. 2021 Jan 22;11:2078. doi: 10.1038/s41598-021-81370-w (PMC7822858; doi:10.1038/s41598-021-81370-w)
Supplement: Supplementary file 1 — Supplementary Information. [file 41598_2021_81370_MOESM1_ESM.pdf]

Mild Synthesis of Isoxazoline Derivatives via An Efficient [4+1] Annulation Reaction of Transient Nitrosoalkenes and Sulfur Ylides

Ting-Bi Hua,[†,1,2] Cheng-Xiong Liu,[†,2] Wei-Min Hu,[1] Long Wang,[1,*] and Qing-Qing Yang,[1,2,*]

1 Key laboratory of inorganic nonmetallic crystalline and energy conversion materials, College of Materials and Chemical Engineering, China Three Gorges University, Yichang, Hubei 443002, China.

2 Hubei Key Laboratory of Natural Products Research and Development, China Three Gorges University, 8 Daxue Road, Yichang, Hubei 443002, China.

* Long Wang: wanglongchem@ctgu.edu.cn; Qing-Qing Yang: qingqing_yang@ctgu.edu.cn

† These authors contributed equally to this work

Supporting Information

Table of Contents

1. General Information S2

2. Reaction Optimization and Result Summary S2-S6

3. References S6

4. Characterizations of Products S7-S13

5. Synthetic Transformation S13

6. Copies of ^{1}H NMR, ^{13}C NMR and ^{19}F NMR Spectra of Compounds **3**. S14-S39

7. Copies of ^{1}H NMR, ^{13}C NMR Spectra of Compound **4aa**. S40

8. Copies of HPLC Chromatograms S41-S42

Experimental Section

1. General Information

General. Unless otherwise noted, materials were purchased from commercial suppliers and used without purification. Dichloromethane was freshly distilled from calcium hydride. Toluene, tetrahydrofuran (THF) and Et_2O were distilled from sodium/benzophenone. Other solvents were also purified before using. Reactions were monitored by thin layer chromatography (TLC), and column chromatography purifications were performed using 200-300 mesh silica gel. ^1H NMR spectra were recorded on 400 MHz spectrophotometers by China Three Gorges University. Solvent for NMR is $CDCl_3$. Chemical shifts are reported in delta (δ) units in parts per million (ppm) relative to the singlet (0 ppm) for tetramethylsilane (TMS), relative to the signal of chloroform (δ 7.26, singlet). Data are reported as follows: chemical shift, multiplicity (s = single, d = doublet, t = triplet, m = multiplet, dd = doublet of doublets), coupling constants (Hz) and integration; ^{13}C NMR spectra were on recorded on 400 (100 MHz) with complete proton decoupling. Chemical shifts are reported in ppm relative to the central line of the heptalet at 77.0 ppm for $CDCl_3$. Melting point was measured with X-4 melting point instrument. High resolution mass spectra (HRMS) analyses were taken on a Shimadzu LCMS-IT-TOF mass spectrometer. IR spectra were recorded on a NEXUS FT-IR spectrometer. Enantiomeric ratios were determined by chiral HPLC with different chiral columns (chiralpak IA-H column) with hexane and i-PrOH as solvents.

Sulfur ylides and α-bromooximes are prepared according to our previous work[1] and references[2].

2. Reaction Optimization and Result Summary

Table S1. The screening of solvents.[a]

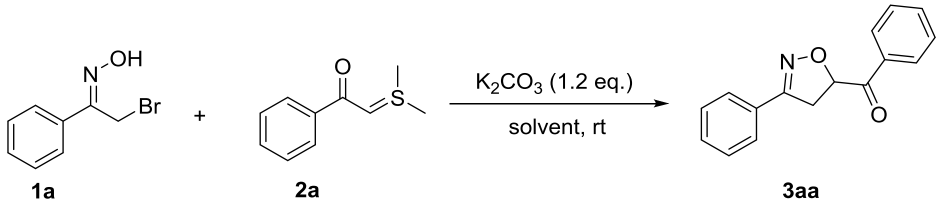

entry	Solvent	yield (%)[b]
1	CH_2Cl_2	71
2	$CHCl_3$	80
3	Et_2O	62
4	DMSO	26
5	toluene	37

[a] Reaction conditions: **1a** (0.40 mmol, 85.6 mg), **2a** (0.48 mmol, 86.5 mg), K_2CO_3 (0.48 mmol, 66.4 mg), solvent (4 mL), rt. [b] Yield of isolated product.

Table S2. The screening of base.[a]

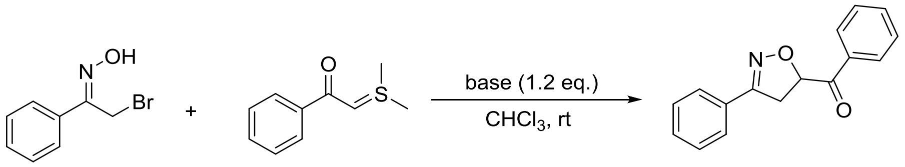

1a **2a** **3aa**

entry	**Base**	yield (%)[b]
1	**K_2CO_3**	80
2	**NaOH**	48
3	***t*-BuOK**	38
4	**Et_3N**	20
5	**Na_2CO_3**	89

[a] Reaction conditions: **1a** (0.40 mmol, 85.6 mg), **2a** (0.48 mmol, 86.5 mg), base (0.48 mmol), $CHCl_3$ (4mL), rt.
[b] Yield of isolated product.

Table S3. The screening of the equivalent of base.[a]

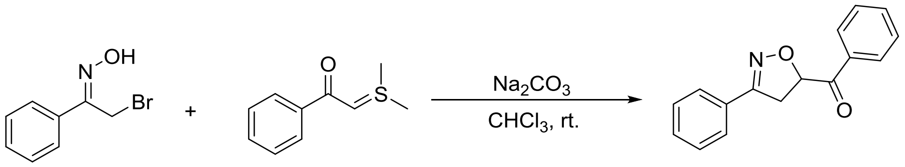

1a **2a** **3aa**

entry	**Equiv.**	Time (h)	yield (%)[b]
1	**1.0**	9.5	89
1	**1.1**	9	91
2	**1.2**	5	89
3	**1.3**	5	91
4	**1.5**	2.5	90

[a] Reaction conditions: **1a** (0.40 mmol, 85.6 mg), **2a** (0.48 mmol, 86.5 mg), Na_2CO_3, $CHCl_3$ (4 mL), rt. [b] Yield of isolated product.

Table S4. The screening of the ratio of substrates.[a]

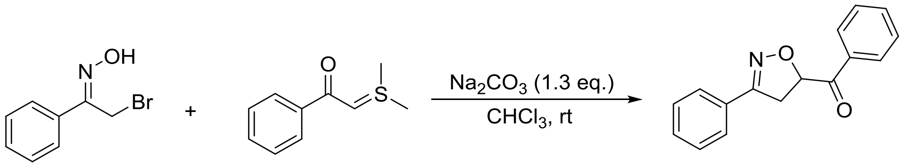

1a **2a** **3aa**

entry	**Ratio**	**Time (h)**	yield (%)[b]

1	**1.0**	**8**	87
2	**1.1**	**6**	89
3	**1.2**	**4**	91
4	**1.3**	**3**	94

[a] Reaction conditions: **1a** (0.40 mmol, 85.6 mg), **2a**, Na_2CO_3 (0.52 mmol, 55.1 mg), $CHCl_3$ (4 mL), rt. [b] Yield of isolated product.

Table S5. The screening of leaving group.[a]

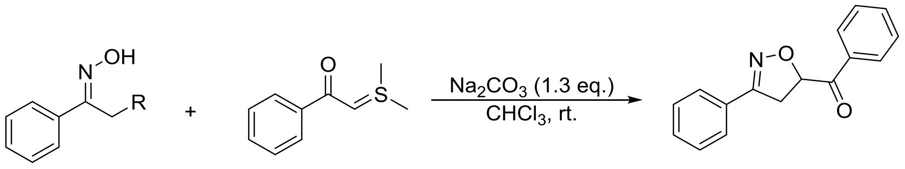

entry	R	yield (%)[b]
1	**Cl**	83
2	**Br**	94

[a] Reaction conditions: **1a** (0.40 mmol, 85.6 mg), **2a** (0.52 mmol), Na_2CO_3 (0.52 mmol, 55.1 mg), $CHCl_3$ (4 mL), rt. [b] Yield of isolated product.

3. References for the Preparation of Substrates

1. For the preparation of sulfur ylide: L.-Q. Lu, Y.-J. Cao, X.-P. Liu, J. An, C.-J. Yao, Z.-H. Ming, W.-J. Xiao, *J. Am. Chem. Soc.* 2008, *130*, 6946-6948.
2. For the preparation of oximes: a) C. Tobias, S. S. Wabnitz, K. A. Jørgensen, *Org. Biomol. Chem.*, 2004, *2*, 828-834; b) M. Voets, I. Antes, C. Scherer, U. Müller-Vieira, K. Biemel, S. Marchais-Oberwinkler, R. W. Hartmann, *J. Med. Chem.*, 2006, *49*, 222-231; c) A. Lemos, J. P. Lourenço, *Tetrahedron Lett.* 2009, *50*, 1311-1313; d) W. Ou, S. Espinosa, H. J. Meléndez, S. M. Farré, J. L. Alvarez, V. Torres, I. Martínez, K. M. Santiago, M. Ortiz-Marciales, *J. Org. Chem.*, 2013, *78*, 5314-5327; e) S. Zhao, H. Wang, S.-F. Sun, H.-B. Guo, Z.-Y. Chen, J. Wang, L. Wang, G.-Q. Wang, *Tetrahedron Lett.*, 2019, *60*, 382-385.

4. Characterizations of Products

Phenyl(3-phenyl-4,5-dihydroisoxazol-5-yl)methanone (3aa).

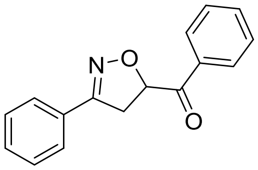

White solid, 94% yield, reaction time for 2 h, mp 100-101 °C. ^1H NMR (400 MHz, $CDCl_3$): δ 8.15-8.08 (m, 2H), 7.75-7.68 (m, 2H), 7.65-7.59 (m, 1H), 7.51 (dd, *J = 10.5, 4.7 Hz*, 2H), 7.46-7.38 (m, 3H), 5.88 (dd, *J = 11.6, 7.1 Hz*, 1H), 4.08 (dd, *J = 16.8, 7.1 Hz*, 1H), 3.54 (dd, *J = 16.8, 11.6 Hz*, 1H); ^{13}C NMR (100 MHz, $CDCl_3$): δ 193.70, 156.76, 134.50, 133.92, 130.43, 129.63 (2C), 128.78, 128.74 (2C), 128.71 (2C), 126.94 (2C), 81.45, 36.06. HRMS: $C_{16}H_{13}NO_2$ [M+Na^+]; calculated: 351.9946, found: 351.9944; IR: 3055, 2967, 1681, 1594, 1446, 1353, 1220, 869, 769, 692 cm^{-1}.

Phenyl(3-(p-tolyl)-4,5-dihydroisoxazol-5-yl)methanone (3ba).

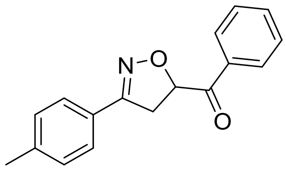

White solid, 92% yield, reaction time for 5 h, mp 103-104 °C. ^{1}H NMR (400 MHz, CDCl3): δ 8.14-8.07 (m, 2H), 7.65-7.57 (m, 3H), 7.54-7.48 (m, 2H), 7.22 (d, *J = 8.0 Hz*, 2H), 5.85 (dd, *J = 11.5, 7.1 Hz*, 1H), 4.05 (dd, *J = 16.8, 7.1 Hz*, 1H), 3.52 (dd, *J = 16.8, 11.5 Hz*, 1H), 2.38 (s, 3H). ^{13}C NMR (100 MHz, CDCl3): δ 193.84, 156.68, 140.71, 134.52, 133.86, 129.61 (2C), 129.42 (2C), 128.68 (2C), 126.87 (2C), 125.93, 81.32, 36.22, 21.46; HRMS: $C_{17}H_{15}NO_2$ [M+H$^+$]; calculated: 266.1176, found: 266.1169; IR: 3066, 2967, 1691, 1593, 1448, 1351, 1232, 881, 692 cm^{-1}.

(4-methoxyphenyl)-4,5-dihydroisoxazol-5-yl)(phenyl)methanone (3ca).

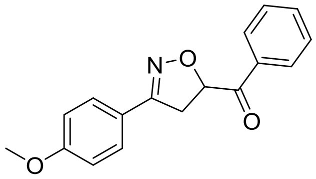

Pale yellow solid, 89% yield, reaction time for 3 h, mp 110-111 °C. ^{1}H NMR (400 MHz, $CDCl_3$): *δ* 8.14-8.08 (m, 2H), 7.68-7.59 (m, 3H), 7.50 (dd, *J = 10.6, 4.7 Hz*, 2H), 6.91 (s, 2H), 5.84 (dd, *J = 11.5, 7.1 Hz*, 1H), 4.04 (dd, *J = 16.7, 7.0 Hz*, 1H), 3.84 (s, 3H), 3.51 (dd, *J = 16.7, 11.5 Hz*, 1H); ^{13}C NMR (100 MHz, $CDCl_3$): *δ* 193.93, 161.27, 156.28, 134.52, 133.84, 129.60 (2C), 128.67 (2C), 128.48 (2C), 121.28, 114.12 (2C), 81.21, 55.32, 36.30; HRMS: $C_{17}H_{15}NO_3$ [M+H$^+$]; calculated: 282.1130, found: 282.1125; IR: 3053, 2968, 1691, 1595, 1352, 1448, 1359, 1230, 881, 763 cm^{-1}.

([1,1'-biphenyl]-4-yl)-4,5-dihydroisoxazol-5-yl)(phenyl)methanone (3da).

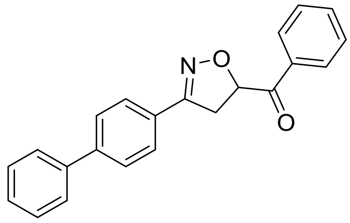

Pale yellow solid, 72% yield, reaction time for 2 h, mp 168-170 °C. ^{1}H NMR (400 MHz, $CDCl_3$): δ 8.18-8.07 (m, 2H), 7.79 (d, *J = 8.4 Hz*, 2H), 7.63 (ddd, *J = 8.8, 5.6, 1.6 Hz*, 5H), 7.52 (t, *J = 7.7 Hz*, 2H), 7.46 (t, *J = 7.5 Hz*, 2H), 7.42-7.34 (m, 1H), 5.90 (dd, *J = 11.5, 7.1 Hz*, 1H), 4.12 (dd, *J = 16.8, 7.1 Hz*, 1H), 3.57 (dd, *J = 16.8, 11.5 Hz*, 1H); ^{13}C NMR (100 MHz, $CDCl_3$): δ 193.69, 156.50, 143.14, 140.07, 134.51, 133.93, 129.64 (2C), 128.89 (2C), 128.72 (2C), 127.86, 127.63, 127.40 (2C), 127.38 (2C), 127.05, 81.50 (2C), 36.08; HRMS: $C_{22}H_{17}NO_2$ [M+H$^+$]; calculated: 328.1332, found: 328.1324; IR: 3056, 2928, 1691, 1595, 1449, 1361, 1232, 913, 839, 763, 692 cm^{-1}.

benzoyl-4,5-dihydroisoxazol-3-yl)benzonitrile (3ea).

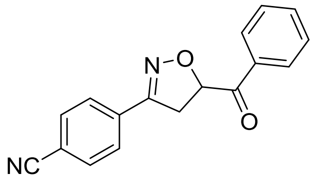

Pale yellow solid, 89% yield, reaction time for 72 h, mp 145-146 ºC. ^{1}H NMR (400 MHz, $CDCl_3$): δ 8.11 (dd, *J = 5.2, 3.3 Hz*, 2H), 7.84-7.80 (m, 2H), 7.73-7.69 (m, 2H), 7.67-7.62 (m, 1H), 7.57-7.51 (m, 2H), 5.97 (dd, *J = 11.6, 7.0 Hz*, 1H), 4.11 (dd, *J = 16.8, 7.0 Hz*, 1H), 3.52 (dd, *J = 16.8, 11.6 Hz*, 1H); ^{13}C NMR (100 MHz, $CDCl_3$): δ 192.85, 155.54, 134.25, 134.17, 133.09, 132.51 (2C), 129.64 (2C), 128.82 (2C), 127.38 (2C), 118.22, 113.77, 81.98, 35.21; HRMS: $C_{17}H_{12}N_2O_2$ [M+Na$^+$]; calculated: 299.0791, found: 299.0794; IR: 3062, 2969, 1682, 1597, 1452, 1351, 1232, 910, 847, 686, 566 cm^{-1}.

(4-fluorophenyl)isoxazol-5-yl)(phenyl)methanone (3fa).

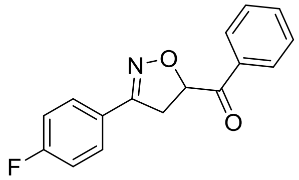

Pale yellow solid, 93% yield, reaction time for 1.5 h, mp 142-143 ºC. ^{1}H NMR (400 MHz, $CDCl_3$): δ 8.14-8.07 (m, 2H), 7.75-7.66 (m, 2H), 7.66-7.58 (m, 1H), 7.51 (dd, *J = 10.6, 4.7 Hz*, 2H), 7.15-7.06 (m, 2H), 5.89 (dd, *J = 11.5, 7.0 Hz*, 1H), 4.11-4.03 (m, 1H), 3.51 (dd, *J = 16.8, 11.5 Hz*, 1H). ^{13}C NMR (100 MHz, $CDCl_3$): δ 193.52, 165.18, 162.68, 155.81, 134.43, 133.97, 129.62 (2C), 128.96, 128.88 , 128.73 (2C), 125.07, 125.04, 116.03, 115.81, 81.45, 77.32, 77.00, 76.68, 36.01; ^{19}F NMR (376 MHz, $CDCl_3$) δ -109.26 (s). HRMS: $C_{16}H_{12}FNO_2$ [M+Na$^+$]; calculated: 292.0744, found: 292.0752; IR: 3052, 2966, 1691, 1605, 1361, 1256, 1174, 827, 746 cm^{-1}.

(2-fluorophenyl)isoxazol-5-yl)(phenyl)methanone (3ga).

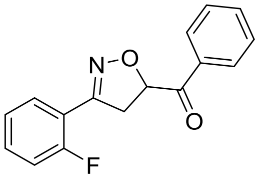

White solid, 90% yield, reaction time for 3 h, mp 92-93 ºC. ^{1}H NMR (400 MHz, $CDCl_3$): δ 8.10 (dd, *J = 5.2, 3.3 Hz*, 2H), 7.85 (td, *J = 7.6, 1.7 Hz*, 1H), 7.70-7.58 (m, 1H), 7.52 (t, *J = 7.6 Hz*, 2H), 7.41 (tdd, *J = 7.2, 5.2, 1.7 Hz*, 1H), 7.22-7.07 (m, 2H), 5.89 (dd, *J = 11.7, 7.2 Hz*, 1H), 4.11 (ddd, *J = 17.6, 7.2, 2.3 Hz*, 1H), 3.67 (ddd, *J = 17.6, 11.7, 2.6 Hz*, 1H); ^{13}C NMR (100 MHz, $CDCl_3$): δ 193.65, 161.70, 159.19, 153.54, 153.51, 134.45, 133.92, 132.12, 132.03, 129.55 (2C), 129.22, 129.19, 128.73 (2C), 124.50, 124.46, 116.97, 116.86, 116.55, 116.34, 81.46, 81.44, 37.91, 37.84; ^{19}F NMR (376 MHz, $CDCl_3$) δ -111.92 (s). HRMS (EI): $C_{16}H_{12}FNO_2$ [M+Na$^+$]; calculated: 292.0744, found: 292.0746; IR: 3072, 2943, 1686, 1594, 1499, 1344, 1229, 891, 762, 688 cm^{-1}.

(3-bromophenyl)isoxazol-5-yl)(phenyl)methanone (3ha).

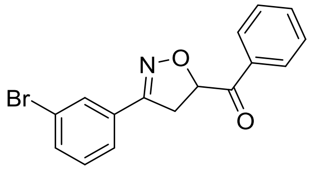

White solid, 99% yield, reaction time for 1.5 h, mp 117-118 ºC. ^{1}H NMR (400 MHz, $CDCl_3$): *δ* 8.15-8.06 (m, 2H), 7.85 (t, *J = 1.7 Hz*, 1H), 7.64 (ddd, *J = 8.8, 2.8, 1.1 Hz*, 2H), 7.54 (ddd, *J = 15.4, 4.6, 1.2 Hz*, 3H), 7.29 (t, *J = 7.9 Hz*, 1H), 5.91 (dd, *J = 11.6, 7.0 Hz*, 1H), 4.06 (dd, *J = 16.8, 7.0 Hz*, 1H), 3.50 (dd, *J = 16.8, 11.6 Hz*, 1H); ^{13}C NMR (100 MHz, $CDCl_3$): *δ* 193.28, 155.65, 134.38, 134.03, 133.32, 130.82, 130.27, 129.86, 129.63 (2C), 128.77 (2C), 125.45, 122.83, 81.63, 35.71; HRMS: $C_{16}H_{12}BrNO_2$ [M+Na$^+$]; calculated: 351.9944, found: 351.9948; IR: 3055, 2970, 1695, 1594, 1448, 1344, 1226, 914, 791, 718, 687 cm^{-1}.

(3-(3,5-bis(trifluoromethyl)phenyl)isoxazol-5-yl)(phenyl)methanone (3ia).

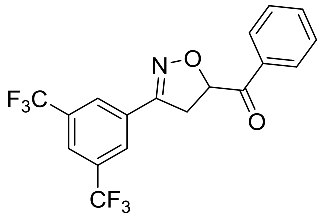

White solid, 72% yield, reaction time for 72 h, mp 98-99 ºC. ^{1}H NMR (400 MHz, $CDCl_3$): δ 8.12 (dd, *J = 12.5, 5.4 Hz*, 4H), 7.93 (s, 1H), 7.70-7.61 (m, 1H), 7.55 (t, *J = 7.7 Hz, 2H*), 6.02 (dd, *J = 11.6, 6.8 Hz*, 1H), 4.15 (dd, *J = 16.8, 6.8 Hz, 1H*), 3.57 (dd, *J = 16.8, 11.6 Hz*, 1H); ^{13}C NMR (100 MHz, $CDCl_3$): δ 192.11, 160.01, 151.70, 134.20 (2C), 134.09, 129.51 (2C), 128.84 (2C), 82.99 (2C), 62.30 (2C), 34.97 (2C), 14.07 (2C); ^{19}F NMR (376 MHz, $CDCl_3$) δ -63.03 (s). HRMS (EI): $C_{18}H_{11}F_6NO_2$ [M+Na$^+$]; calculated: 410.0586, found: 410.0586; IR: 3096, 2932, 1687, 1598, 1452, 1320, 1278, 1131, 898, 705 cm^{-1}.

(3-(furan-2-yl)isoxazol-5-yl)(phenyl)methanone (3ja).

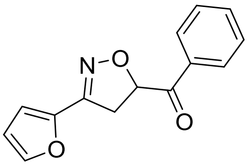

White solid, 76% yield, reaction time for 2 h, mp 93-94 ºC. ^{1}H NMR (400 MHz, $CDCl_3$): *δ* 8.10 (d, *J = 7.4 Hz*, 2H), 7.63 (t, *J = 7.4 Hz*, 1H), 7.56-7.48 (m, 3H), 6.80 (d, J = 3.4 Hz, 1H), 6.51 (dd, J = 3.4, 1.8 Hz, 1H), 5.84 (dd, *J = 11.5, 7.1 Hz*, 1H), 4.05 (dd, *J = 16.9, 7.1 Hz*, 1H), 3.51 (dd, *J = 16.9, 11.5 Hz*, 1H); ^{13}C NMR (100 MHz, $CDCl_3$): *δ* 193.36, 148.93, 144.64, 144.12, 134.46, 133.96, 129.63 (2C), 128.73 (2C), 112.59, 111.79, 81.04, 35.96; HRMS (EI): $C_{14}H_{11}NO_3$ [M+Na$^+$]; calculated: 264.0631, found: 264.0630; IR: 3149, 2953, 1688, 1596, 1449, 1344, 1230, 1003, 867, 687 cm^{-1}.

(3-(tert-butyl)isoxazol-5-yl)(phenyl)methanone (3ka).

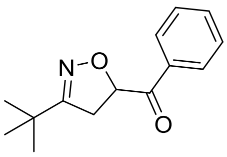

Light yellow oil, 99% yield, reaction time for 2 h. ^{1}H NMR (400 MHz, $CDCl_3$): δ 8.11- 8.04 (m, 2H), 7.60 (t, *J = 7.4 Hz*, 1H), 7.49 (t, *J = 7.6 Hz*, 2H), 5.67 (dd, *J = 11.2, 6.7 Hz*, 1H), 3.67 (dd, *J = 16.9, 6.7 Hz*, 1H), 3.15 (dd, *J = 16.9, 11.2 Hz*, 1H), 1.24 (s, 9H); ^{13}C NMR (100 MHz, $CDCl_3$): δ 194.17, 166.28, 134.57, 133.73, 129.56 (2C), 128.60 (2C), 80.59, 35.44, 33.09, 28.08 (2C), 27.74; HRMS: $C_{14}H_{17}NO_2$ [$M+H^+$]; calculated: 232.1332, found: 232.1337; IR: 3063, 2969, 1691, 1596, 1452, 1365, 1228, 867, 690 cm^{-1}.

ethyl 5-benzoyl-4,5-dihydroisoxazole-3-carboxylate (3la).

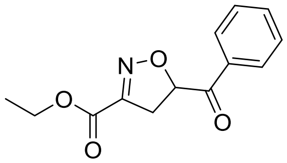

Yellow oil, 89% yield, reaction time for 1.5 h. ^{1}H NMR (400 MHz, $CDCl_3$): δ 8.06 (d, *J = 7.4 Hz*, 2H), 7.64 (t, *J = 7.4 Hz*, 1H), 7.52 (t, *J = 7.8 Hz*, 2H), 5.95 (dd, *J = 12.1, 7.5 Hz*, 1H), 4.37 (q, *J = 7.1 Hz*, 2H), 3.92 (dd, *J = 17.9, 7.5 Hz*, 1H), 3.42 (dd, *J = 17.9, 12.1 Hz*, 1H), 1.37 (t, *J = 7.1 Hz*, 3H); ^{13}C NMR (100 MHz, $CDCl_3$): δ 192.11, 160.01, 151.70, 134.20, 134.09, 129.51 (2C), 128.84 (2C), 82.99, 62.30, 34.97, 14.07; HRMS: $C_{13}H_{13}NO_3$ [$M+Na^+$]; calculated: 270.0737, found: 270.0733; IR: 3067, 2985, 1695, 1598, 1450, 1380, 1230, 1129, 1019, 913, 692 cm^{-1}.

Phenyl(3,3a,4,5-tetrahydronaphtho[1,2-c]isoxazol-3-yl)methanone (3ma).

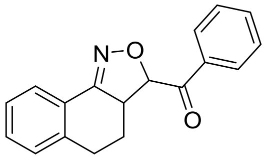

Yellow solid, 87% yield, *dr* > 95 :5, reaction time for 1 h, mp 99-100 ^oC. ^{1}H NMR (400 MHz, $CDCl_3$): δ 8.18-8.11 (m, 2H), 7.95 (d, *J = 7.8 Hz*, 1H), 7.63 (t, *J = 7.4 Hz*, 1H), 7.52 (t, *J = 7.7 Hz*, 2H), 7.36 (td, *J = 7.5, 1.3 Hz*, 1H), 7.27 (dd, *J = 8.4, 5.7 Hz*, 2H), 5.34 (d, *J = 12.1 Hz*, 1H), 4.05 (td, *J = 12.7, 4.9 Hz*, 1H), 3.13-2.96 (m, 2H), 2.35 (dtd, *J = 12.1, 4.8, 2.3 Hz*, 1H), 1.94 (qd, *J = 12.7, 5.1 Hz*, 1H); ^{13}C NMR (100 MHz, $CDCl_3$): δ 193.80, 158.17, 138.92, 135.03, 134.03, 130.77, 129.66 (2C), 129.02, 128.70 (2C), 126.84, 125.55, 124.81, 87.85, 49.31, 29.30, 27.48; HRMS (EI): $C_{18}H_{15}NO_2$ [$M+Na^+$]; calculated: 300.0995, found: 300.0991; IR: 3065, 2929, 1687, 1597, 1451, 1350, 1255, 862, 768, 696 cm^{-1}.

(4-phenyl-4,5-dihydroisoxazol-5-yl)(p-tolyl)methanone (3ab).

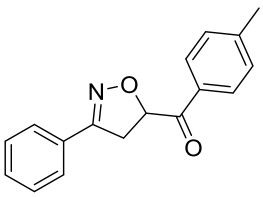

White solid, 99% yield, reaction time for 2 h, mp 118-119 ºC. ^{1}H NMR (400 MHz, $CDCl_3$): δ 8.01 (d, *J = 8.2 Hz*, 2H), 7.75-7.67 (m, 2H), 7.45-7.37 (m, 3H), 7.31 (d, *J = 8.0 Hz*, 2H), 5.86 (dd, *J = 11.6, 7.2 Hz*, 1H), 4.07 (dd, *J = 16.8, 7.2 Hz*, 1H), 3.52 (dd, *J = 16.8, 11.6 Hz*, 1H), 2.43 (s, 3H); ^{13}C NMR (100 MHz, $CDCl_3$): δ 193.32, 156.78, 145.01, 132.09, 130.44, 129.79 (2C), 129.49 (2C), 128.91, 128.78 (2C), 126.99 (2C), 81.50, 36.10, 21.83; HRMS: $C_{17}H_{15}NO_2$ [M+H^{+}]; calculated: 266.1176, found: 266.1167; IR: 3056, 2953, 1680, 1606, 1356, 1233, 890, 763 cm^{-1}.

(5-methoxyphenyl)(3-phenyl-4,5-dihydroisoxazol-5-yl)methanone (3ac).

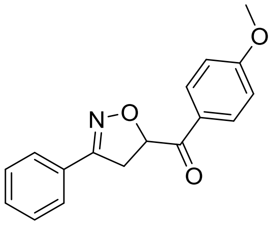

Light yellow solid, 89% yield, reaction time for 8 h, mp 123-124 ºC. ^{1}H NMR (400 MHz, $CDCl_3$): δ 8.11 (d, *J = 9.0 Hz*, 2H), 7.76-7.66 (m, 2H), 7.42 (dd, *J = 5.2, 1.9 Hz*, 3H), 6.98 (d, *J = 9.0 Hz*, 2H), 5.84 (dd, *J = 11.5, 7.3 Hz*, 1H), 4.09 (dd, *J = 16.8, 7.3 Hz*, 1H), 3.89 (s, 3H), 3.51 (dd, *J = 16.8, 11.5 Hz*, 1H); ^{13}C NMR (100 MHz, $CDCl_3$): δ 192.16, 164.19, 156.86, 132.10 (2C), 130.42, 128.94, 128.78 (2C), 127.63, 126.99 (2C), 114.01 (2C), 81.54, 55.59, 36.13; HRMS: $C_{17}H_{15}NO_3$ [M+Na^{+}]; calculated: 304.0944, found: 304.0951; IR: 3004, 2967, 1668, 1599, 1358, 1237, 1182, 879, 763 cm^{-1}.

(4-chlorophenyl)(3-phenyl-4,5-dihydroisoxazol-5-yl)methanone (3ad).

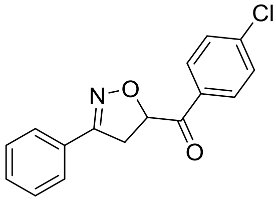

Brown solid, 87% yield, reaction time for 8.5 h, mp 118-119 ºC. ^{1}H NMR (400 MHz, $CDCl_3$): δ 8.12-8.02 (m, 2H), 7.76-7.66 (m, 2H), 7.54-7.46 (m, 2H), 7.46-7.38 (m, 3H), 5.82 (dd, *J = 11.5, 7.0 Hz*, 1H), 4.10 (dd, *J = 16.9, 7.0 Hz*, 1H), 3.53 (dd, *J = 16.9, 11.5 Hz*, 1H); ^{13}C NMR (100 MHz, $CDCl_3$): δ 192.69, 156.90, 140.49, 132.85, 131.09 (2C), 130.53, 129.06 (2C), 128.78 (2C), 128.64, 126.96 (2C), 81.53, 35.88; HRMS: $C_{16}H_{12}ClNO_2$ [M+H^{+}]; calculated: 286.0629, found: 286.0633; IR: 3064, 2961, 1591, 1355, 1226, 888, 756, 688 cm^{-1}.

(4-bromophenyl)(3-phenyl-4,5-dihydroisoxazol-5-yl)methanone (3ae).

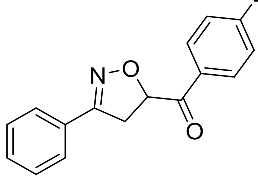

Light grey solid, 96% yield, reaction time for 3 h, mp 130-131 ºC. ^{1}H NMR (400 MHz, $CDCl_3$): δ 7.99 (d, *J = 8.6 Hz*, 2H), 7.75-7.62 (m, 4H), 7.49- 7.35 (m, 3H), 5.81 (dd, *J = 11.5, 7.0 Hz*, 1H), 4.10

(dd, *J = 16.9, 6.9 Hz*, 1H), 3.53 (dd, *J = 16.9, 11.5 Hz*, 1H); ^{13}C NMR (100 MHz, $CDCl_3$): δ 192.93, 156.91, 133.26, 132.07 (2C), 131.16 (2C), 130.55, 129.33 (2C), 128.79, 128.63, 126.96 (2C), 81.53, 35.90; HRMS (EI): $C_{16}H_{12}BrNO_2$ [M^++Na^+]; calculated: 351.9944, found: 351.9946; IR: 3079, 2960, 1699, 1588, 1456, 1359, 1229, 862, 757, 692 cm^{-1}.

(3-bromophenyl)(3-phenyl-4,5-dihydroisoxazol-5-yl)methanone (3af).

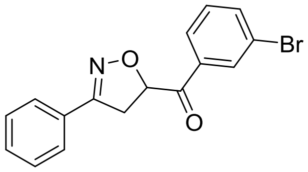

Yellow solid, 92% yield, reaction time for 1.5 h, mp 104-105 °C. ^1H NMR (400 MHz, $CDCl_3$): δ 8.25 (t, *J = 1.7 Hz*, 1H), 8.06 (d, *J = 7.8 Hz*, 1H), 7.79-7.66 (m, 3H), 7.48-7.36 (m, 4H), 5.82 (dd, *J = 11.5, 6.9 Hz*, 1H), 4.10 (dd, *J = 16.9, 6.9 Hz*, 1H), 3.54 (dd, *J = 16.9, 11.5 Hz*, 1H); ^{13}C NMR (100 MHz, $CDCl_3$): δ 192.60, 156.89, 136.77, 136.25, 132.58, 130.57, 130.28, 128.80 (2C), 128.61, 128.26, 126.98 (2C), 123.03, 81.44, 35.89; HRMS: $C_{16}H_{12}BrNO_2$ [M+Na^+]; calculated: 351.9944, found: 351.9947; IR: 3061, 2927, 1695, 1565, 1447, 1354, 1226, 922, 758, 696 cm^{-1}.

Naphthalen-2-yl(3-phenyl-4,5-dihydroisoxazol-5-yl)methanone (3ag).

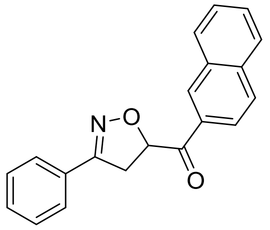

Light red solid, 94% yield, reaction time for 1 h, mp 154-155 °C. ^1H NMR (400 MHz, $CDCl_3$): δ 8.70 (s, 1H), 8.12 (dd, *J = 8.6, 1.7 Hz*, 1H), 8.01 (d, *J = 8.0 Hz*, 1H), 7.91 (dd, *J = 15.8, 8.4 Hz*, 2H), 7.79-7.67 (m, 2H), 7.60 (dtd, *J = 16.2, 6.9, 1.2 Hz*, 2H), 7.47-7.36 (m, 3H), 6.02 (dd, *J = 11.5, 7.2 Hz*, 1H), 4.16 (dd, *J = 16.8, 7.2 Hz*, 1H), 3.58 (dd, *J = 16.8, 11.5 Hz*, 1H); ^{13}C NMR (100 MHz, $CDCl_3$): δ 193.57, 156.86, 135.93, 132.44, 132.16, 131.86, 130.44, 129.91, 128.97, 128.81, 128.75 (2C), 128.59, 127.79, 126.96 (2C), 126.89, 124.56, 81.58, 36.14; HRMS: $C_{20}H_{15}NO_2$ [M+Na^+]; calculated: 324.0995, found: 324.0993; IR: 3054, 2983, 1674, 1358, 1223, 883, 768, 750, 697 cm^{-1}.

Furan-2-yl(3-phenyl-4,5-dihydroisoxazol-5-yl)methanone (3ah).

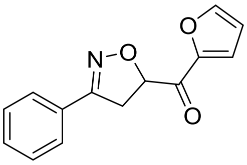

Yellow solid, 76% yield, reaction time for 5 h, mp 56-57 °C. ^1H NMR (400 MHz, $CDCl_3$): δ 7.70 (dd, *J = 7.5, 2.0 Hz*, 3H), 7.59 (d, *J = 3.6 Hz*, 1H), 7.42 (dd, *J = 5.9, 4.4 Hz*, 3H), 6.61 (dd, *J = 3.6, 1.7 Hz*, 1H), 5.64 (dd, *J = 11.7, 6.8 Hz*, 1H), 3.94 (dd, *J = 16.9, 6.8 Hz*, 1H), 3.61 (dd, *J = 16.9, 11.7 Hz*, 1H); ^{13}C NMR (100 MHz, $CDCl_3$): δ 183.68, 156.62, 150.38, 147.83, 130.51, 128.76 (2C), 128.56, 126.94 (2C), 121.48, 112.63, 81.66, 37.10; HRMS: $C_{14}H_{11}NO_3$ [M+Na^+]; calculated: 264.0631, found:

264.0630; IR: 3134, 2936, 1670, 1462, 1391, 1356, 1268, 1025, 875, 761, 690 cm^{-1}.

2,2-dimethyl-1-(3-phenyl-4,5-dihydroisoxazol-5-yl)propan-1-one (3ai).

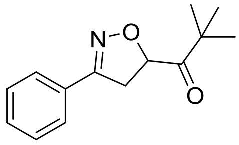

Yellow solid, 51% yield, reaction time for 5 h, use sulfur ylide 4.0 eq., mp 54-56 °C. ^1H NMR (400 MHz, $CDCl_3$): δ 7.69 – 7.62 (m, 2H), 7.42 – 7.34 (m, 3H), 5.41 (dd, J = 11.5, 7.5 Hz, 1H), 3.70 (dd, J = 16.7, 7.5 Hz, 1H), 3.39 (dd, J = 16.7, 11.4 Hz, 1H), 1.26 (s, 9H); ^{13}C NMR (100 MHz, $CDCl_3$): δ 210.05, 156.23 , 130.13 , 128.57 (2C), 126.67 (2C), 80.27, 77.32, 77.00, 76.68, 43.66, 37.09, 26.03 (3C).; HRMS: $C_{14}H_{17}NO_2$ [M+H$^+$]; calculated: 232.1332 , found: 232.1333; IR: 3115, 2928, 1669, 1593, 1443, 1361, 1248, 825, 690 cm^{-1}.

5. Synthetic Transformation

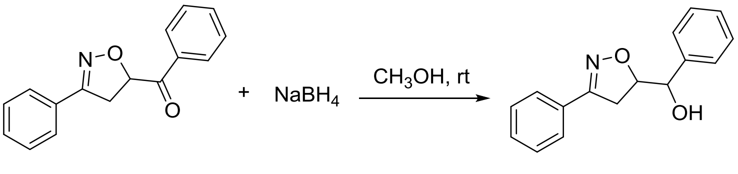

General procedure for the Synthetic transformation of isoxazoline derivative **3aa**. To a solution of **3aa** (0.5 mmol) in methanol (1.0 mL), $NaBH_4$ (0.5 mmol) was added. The reaction was stirring at room temperature for 20 min. The reaction was monitored via TLC (petroleum ether/ethyl acetate = 2:1). Upon consumption of the starting material, the reaction mixture was purified by flash chromatography on silica gel (petroleum ether/ethyl acetate = 10:1) to give the desired product **4aa** in 90% yield with 3:2 dr value.

Phenyl(3-phenyl-4,5-dihydroisoxazol-5-yl)methanol (4aa)

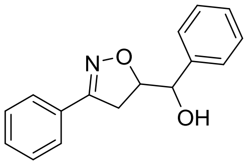

White solid, 90% yield, *dr* = 3:2, reaction time for 20 min, mp 155-156 °C. ^1H NMR (400 MHz, $CDCl_3$): δ 7.67-7.59 (m, 2H), 7.45-7.31 (m, 8H), 4.91 (dt, J = *10.6, 7.0 Hz*, 1H), 4.64 (d, J = *7.3 Hz*, 1H), 3.25 (dd, J = *17.0, 10.6 Hz*, 1H), 3.09 (dd, J = *17.0, 6.8 Hz*, 1H), 2.91 (dd, J = *19.3, 9.0 Hz*, 1H); ^{13}C NMR (100 MHz, $CDCl_3$): δ 156.99, 139.02, 130.31, 129.06, 128.74 (2C), 128.71 (2C), 128.53, 127.22 (2C), 126.71 (2C), 84.60, 75.89, 37.19; HRMS: $C_{16}H_{15}NO_2$ [M+Na$^+$]; calculated: 276.0995, found: 276.0997; IR: 3475, 2058, 2948, 1599, 1451, 1363, 1093, 902, 746, 702, 693 cm^{-1}.

6. ^{1}H NMR, ^{13}C NMR and ^{19}F NMR spectra of compounds 3.

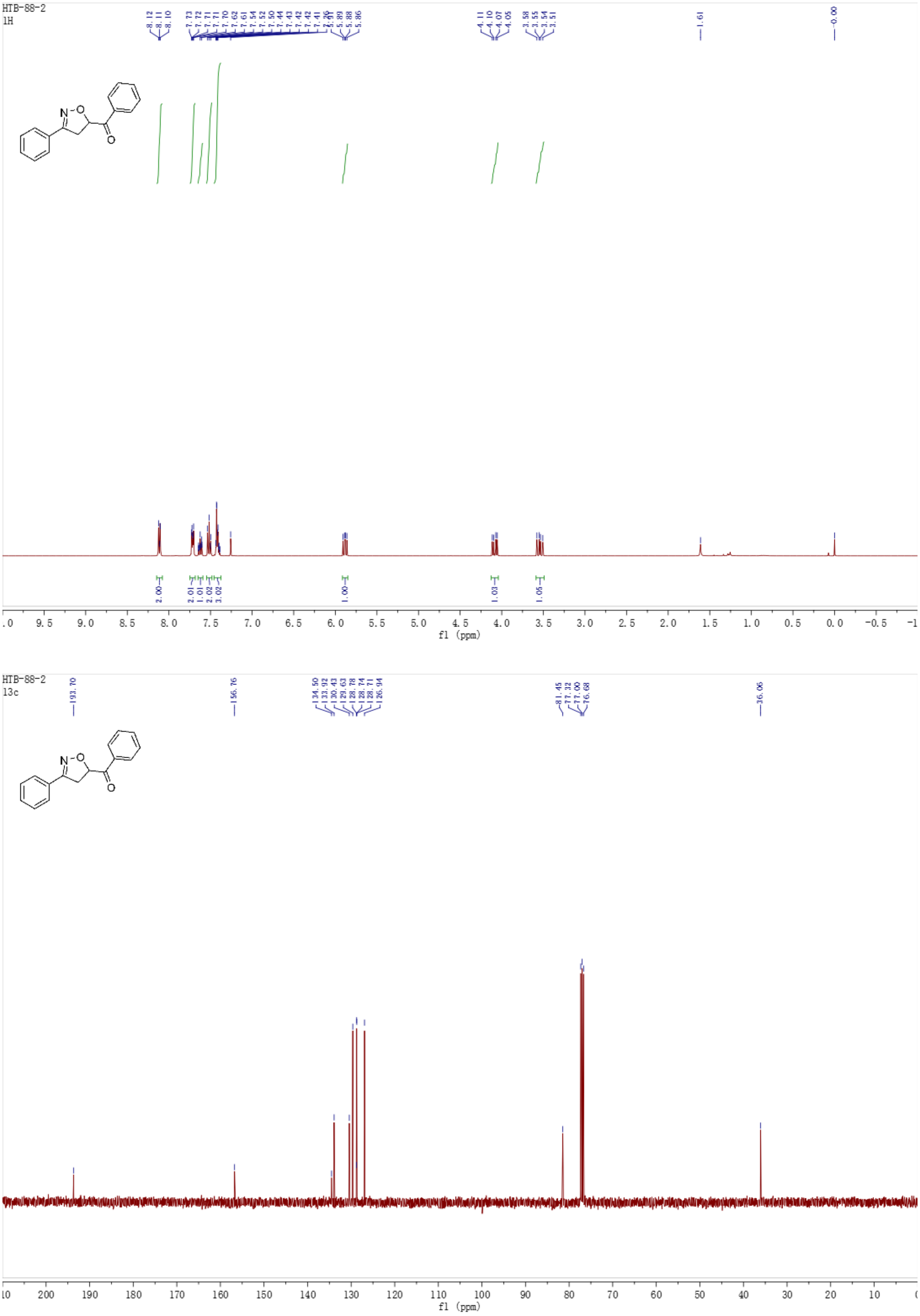

Figure S1. The ^{1}H NMR and ^{13}C NMR Spectrum of **3aa** in $CDCl_3$.

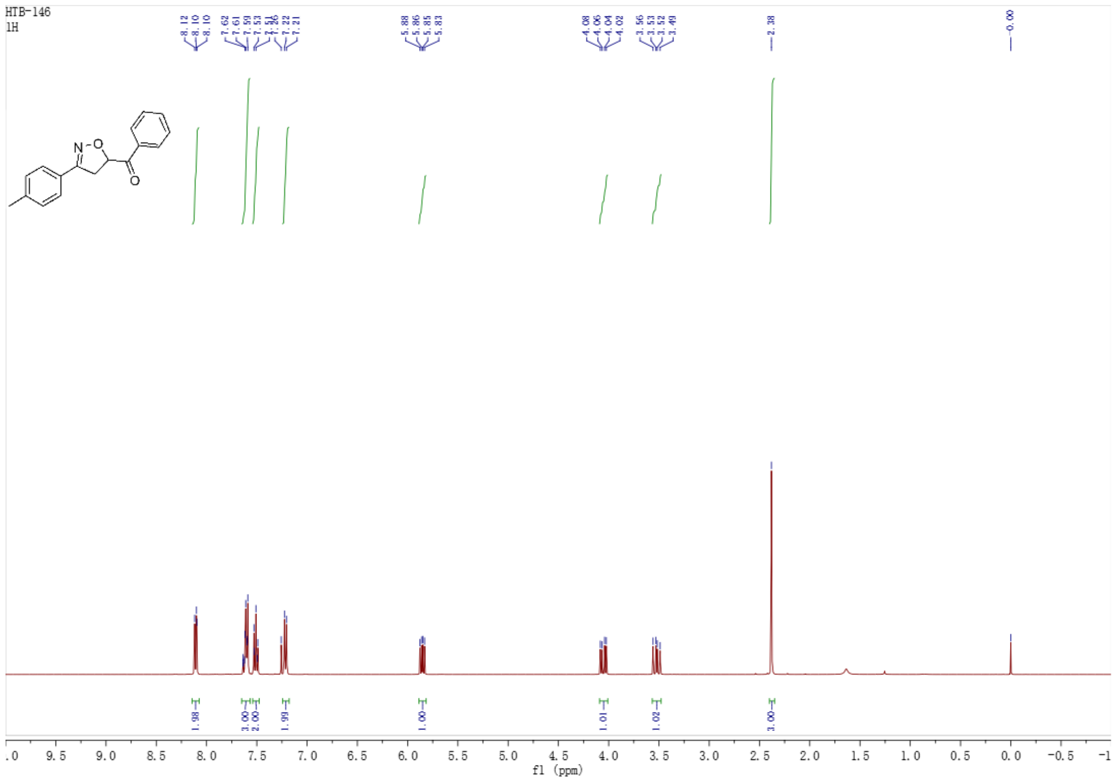

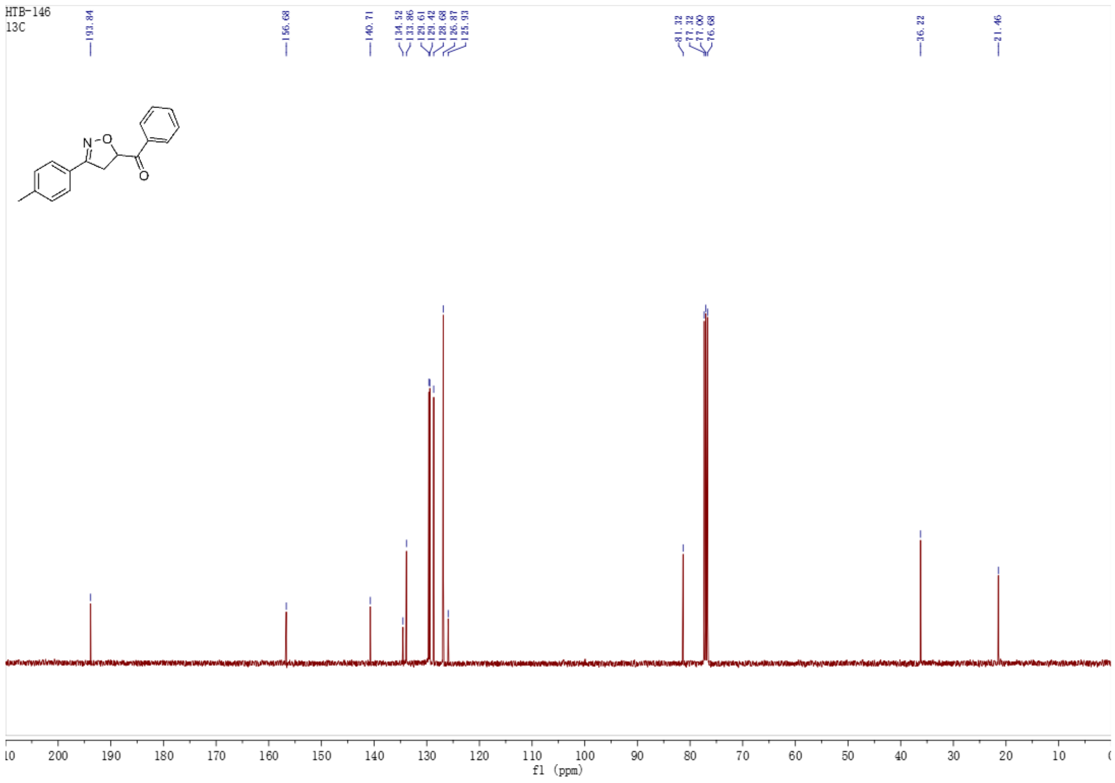

Figure S2. The ^{1}H NMR and ^{13}C NMR Spectrum of **3ba** in $CDCl_3$.

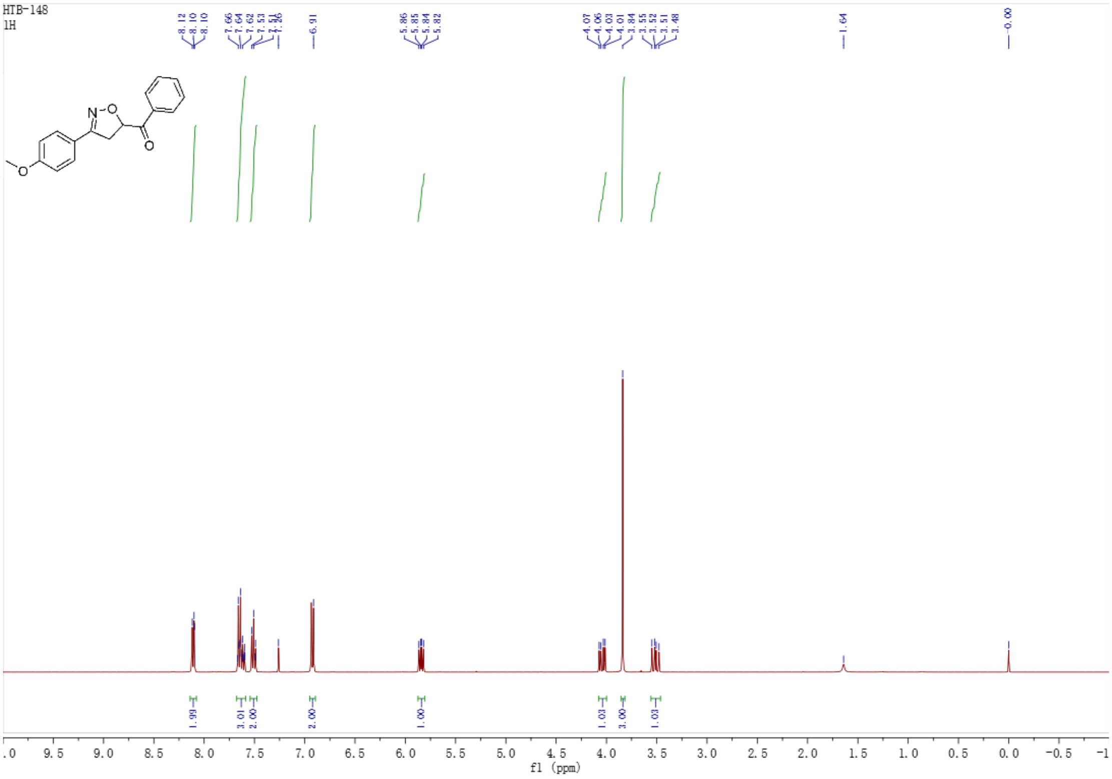

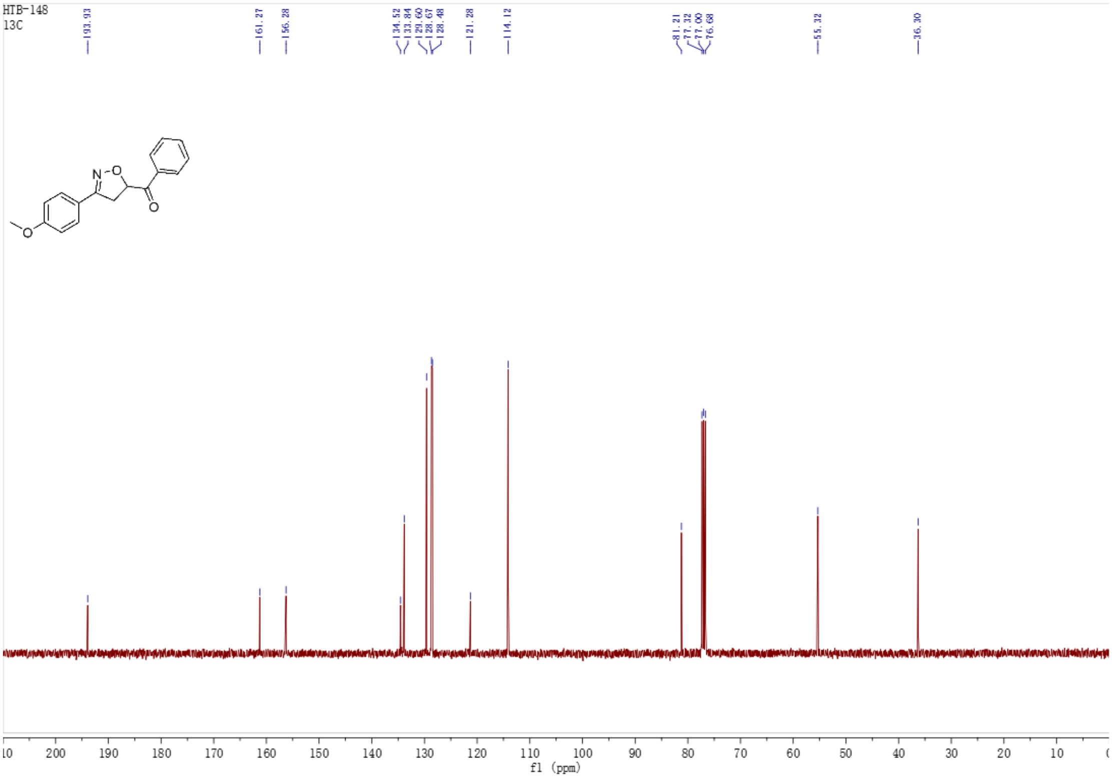

Figure S3. The ^{1}H NMR and ^{13}C NMR Spectrum of **3ca** in $CDCl_3$.

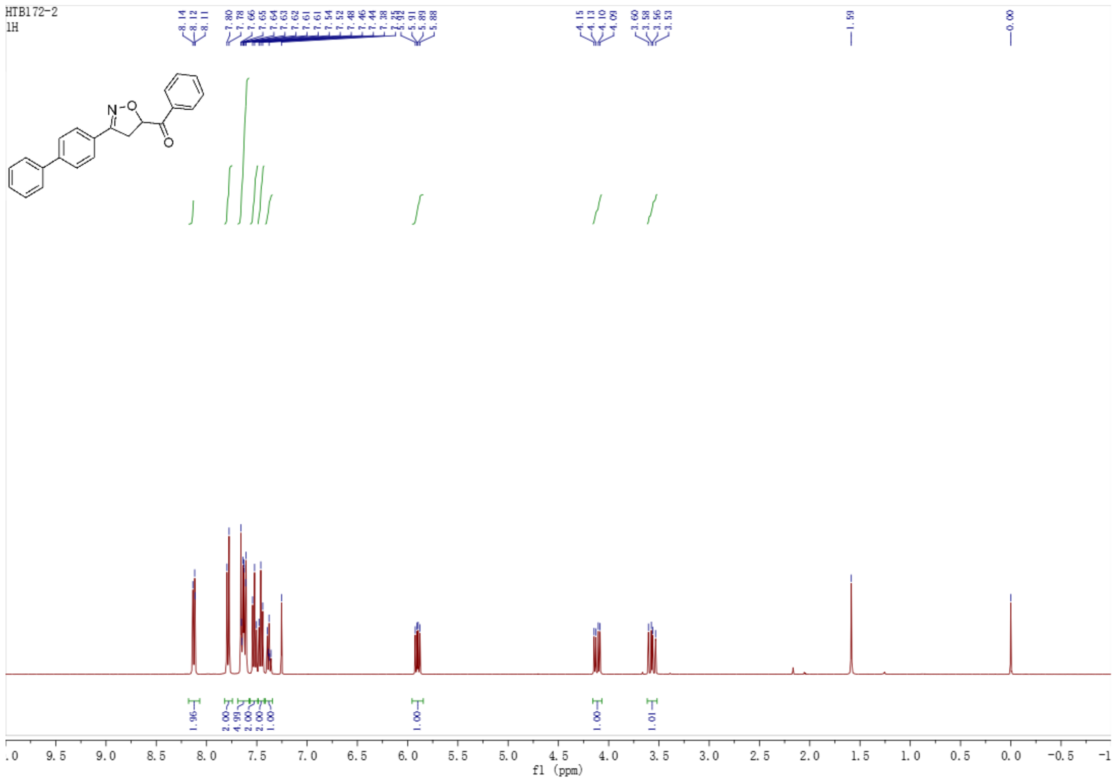

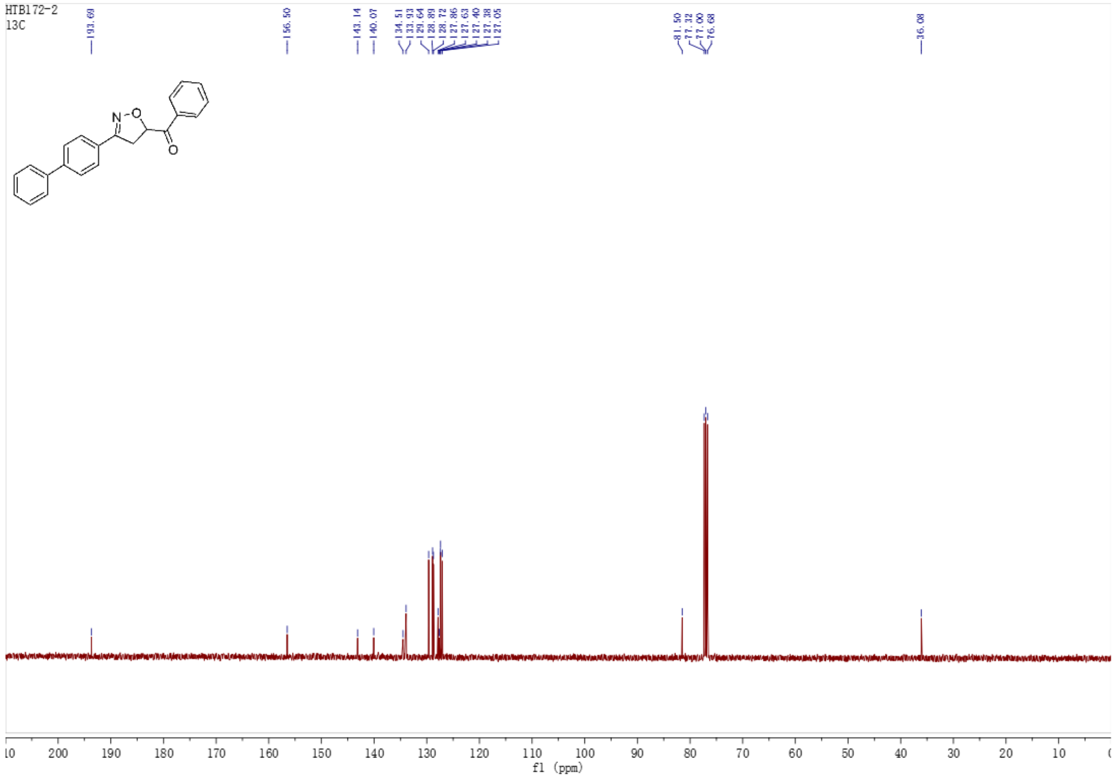

Figure S4. The ^{1}H NMR and ^{13}C NMR Spectrum of **3da** in $CDCl_3$.

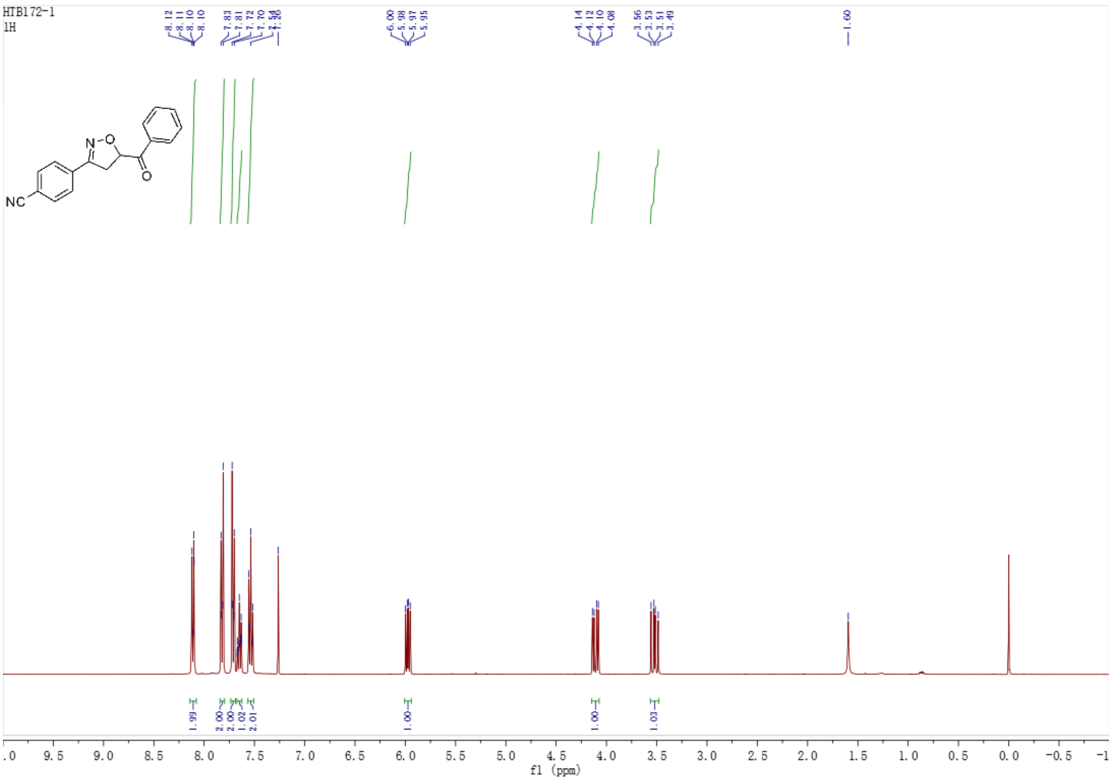

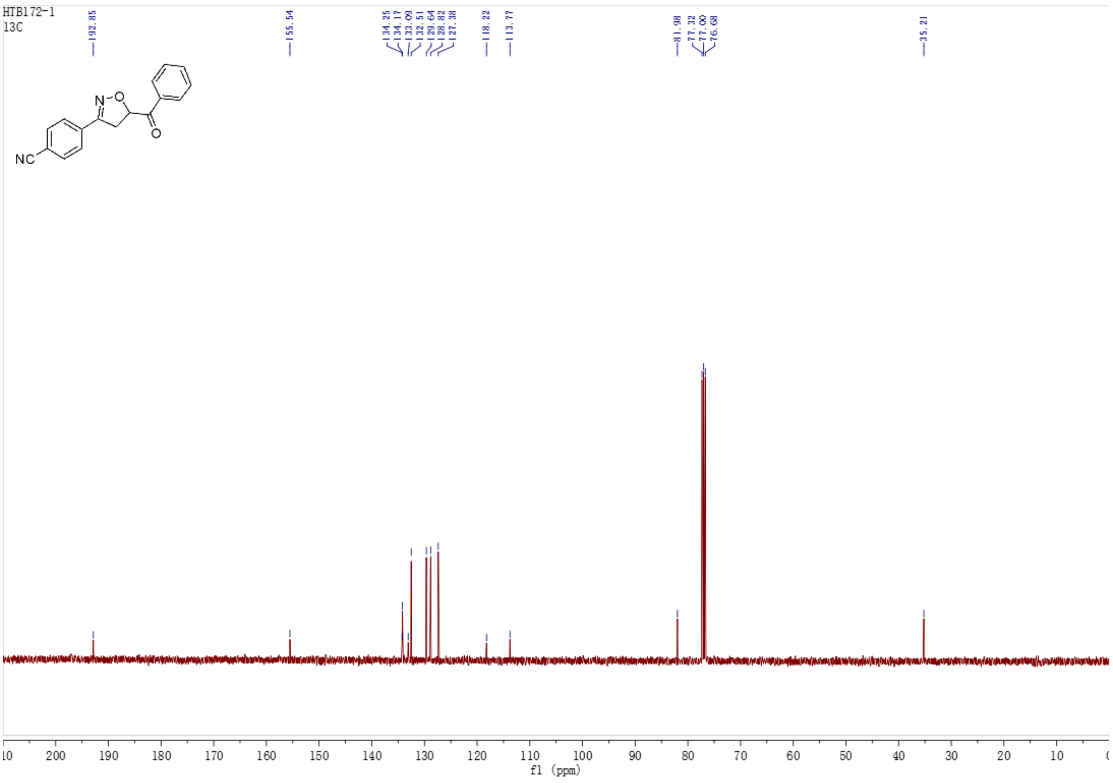

Figure S5. The ^{1}H NMR and ^{13}C NMR Spectrum of **3ea** in $CDCl_3$.

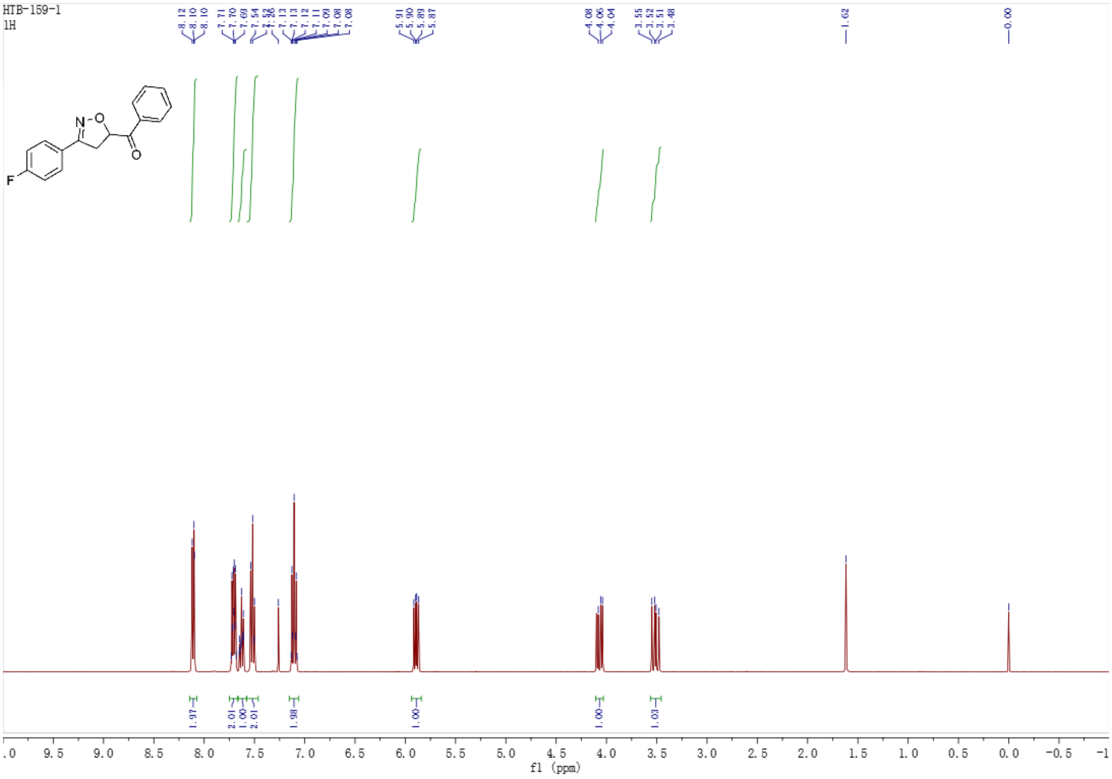

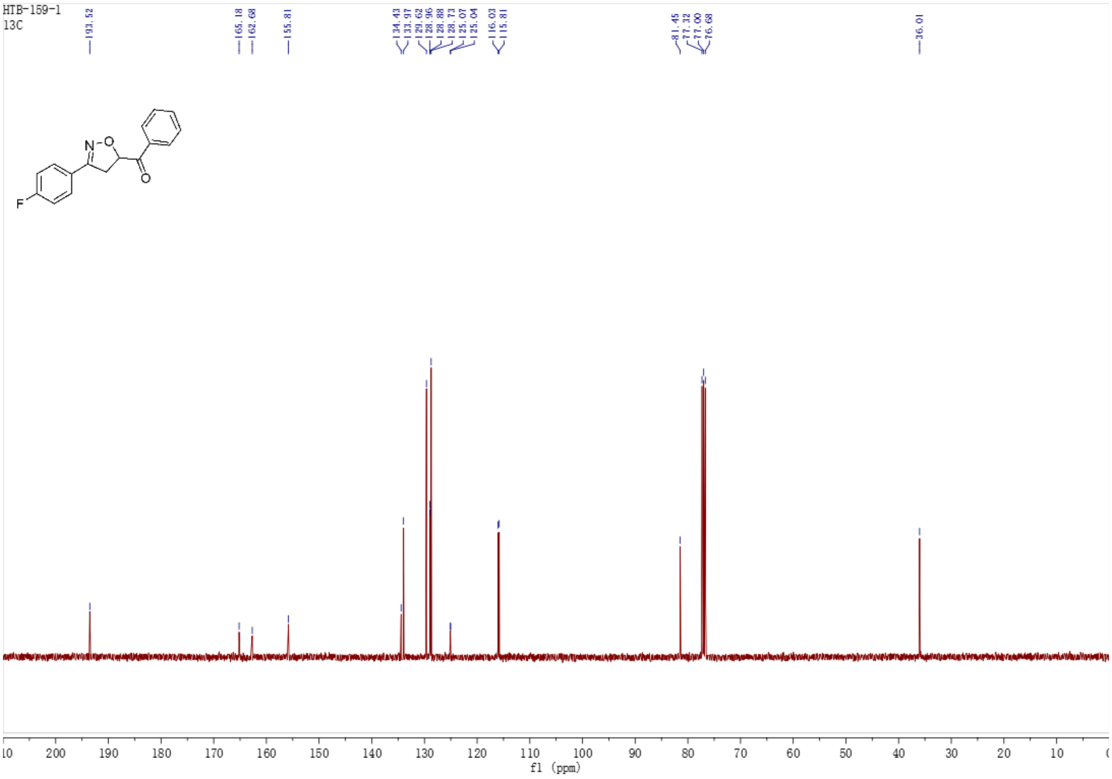

Figure S6-1. The ^{1}H NMR and ^{13}C NMR Spectrum of **3fa** in $CDCl_3$.

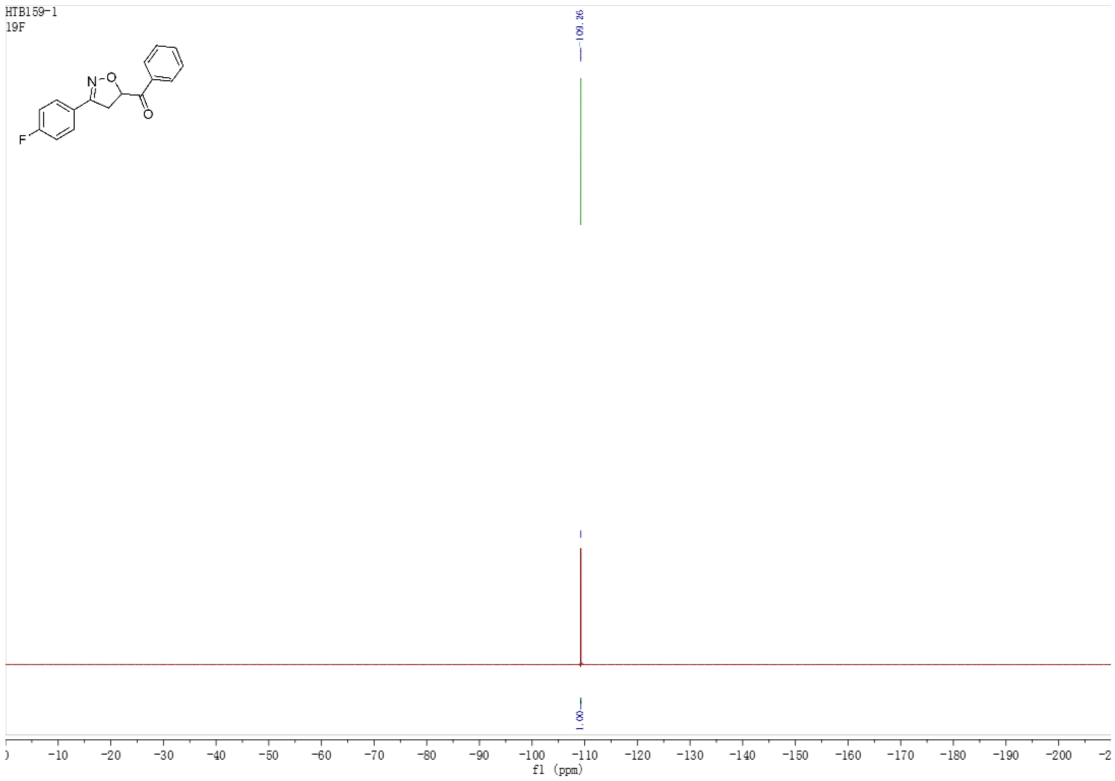

Figure S6-2. The ^{19}F NMR Spectrum of **3fa** in $CDCl_3$.

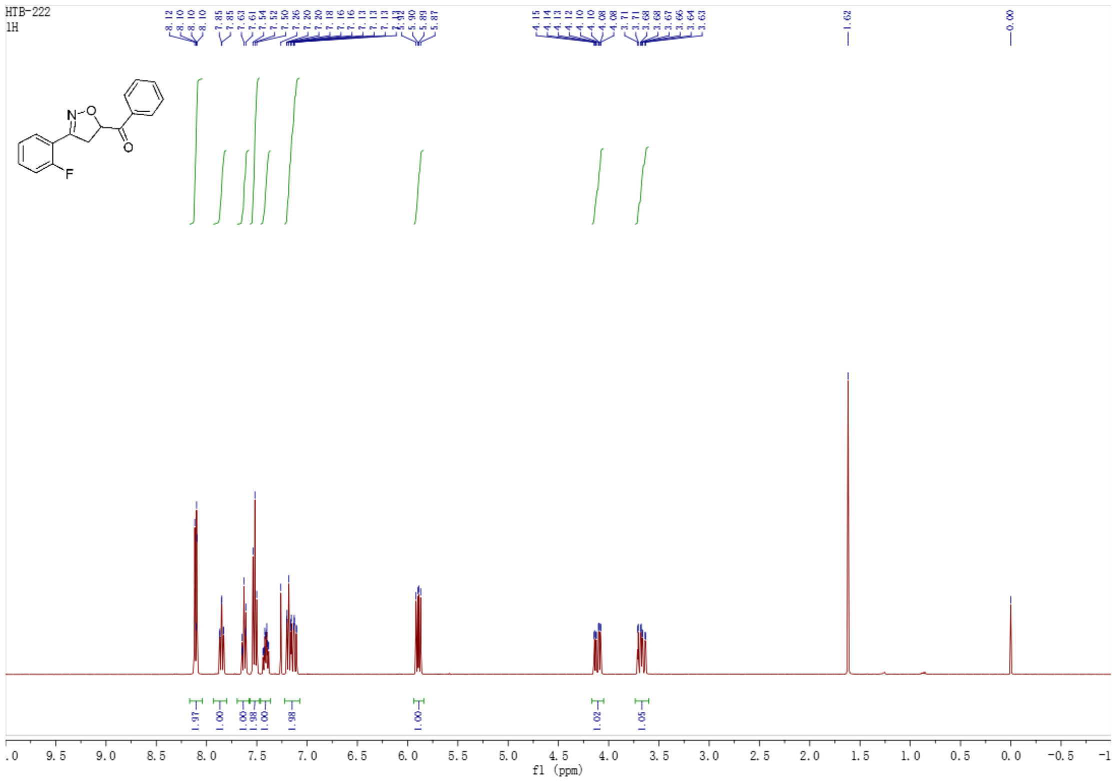

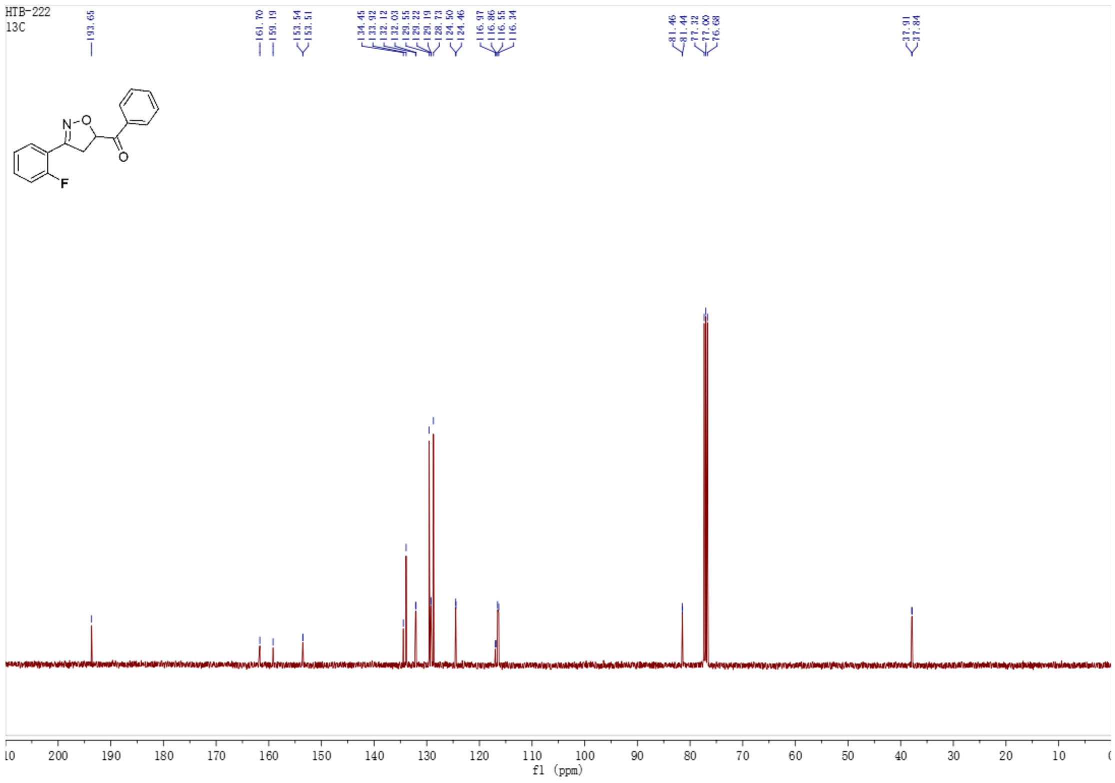

Figure S7-1. The ^{1}H NMR and ^{13}C NMR Spectrum of **3ga** in $CDCl_3$.

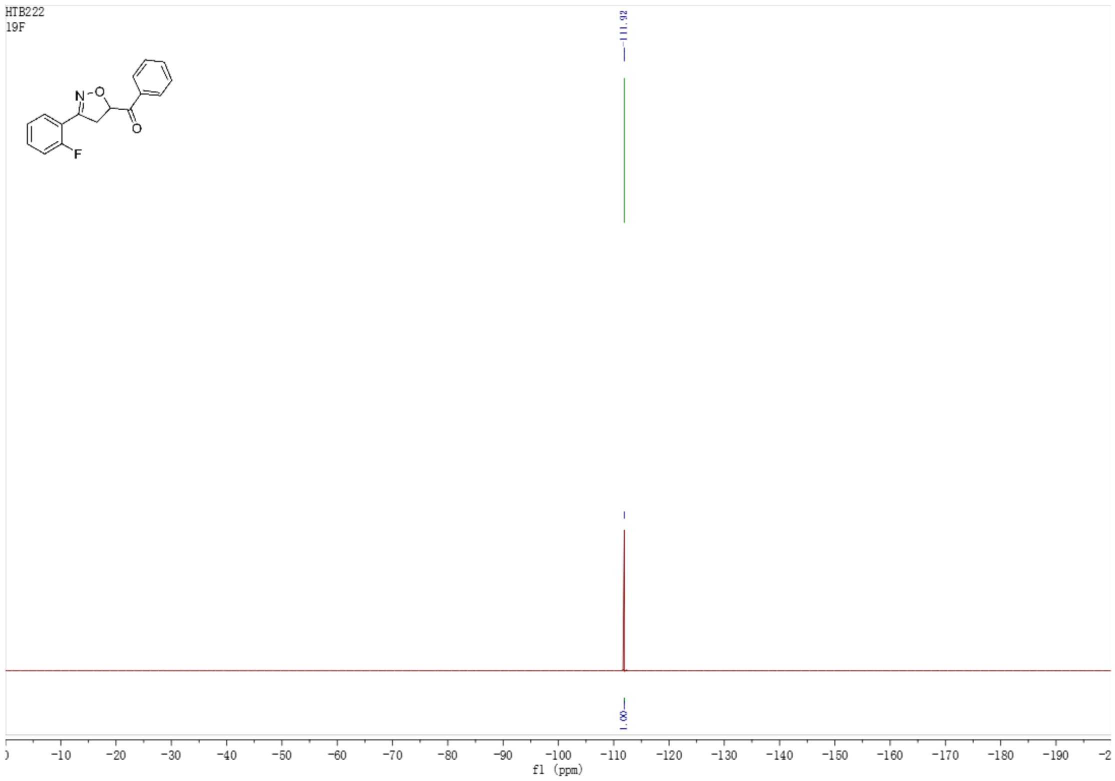

Figure S7-2. The ^{19}F NMR Spectrum of **3ga** in $CDCl_3$.

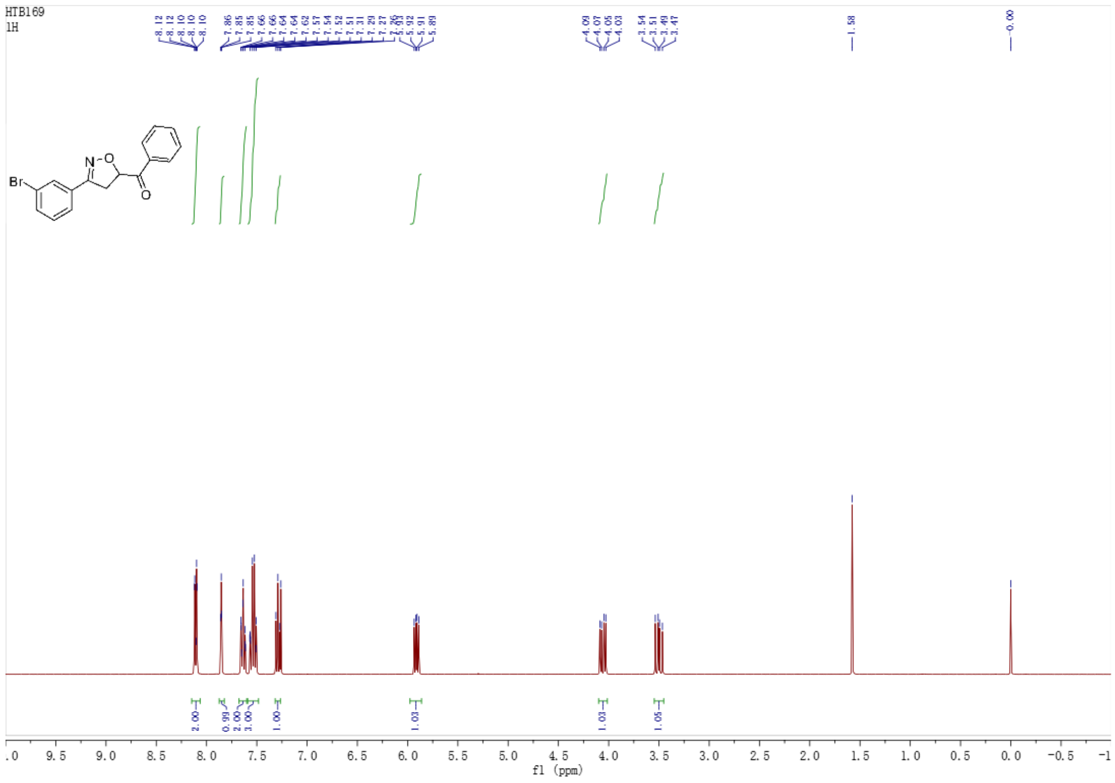

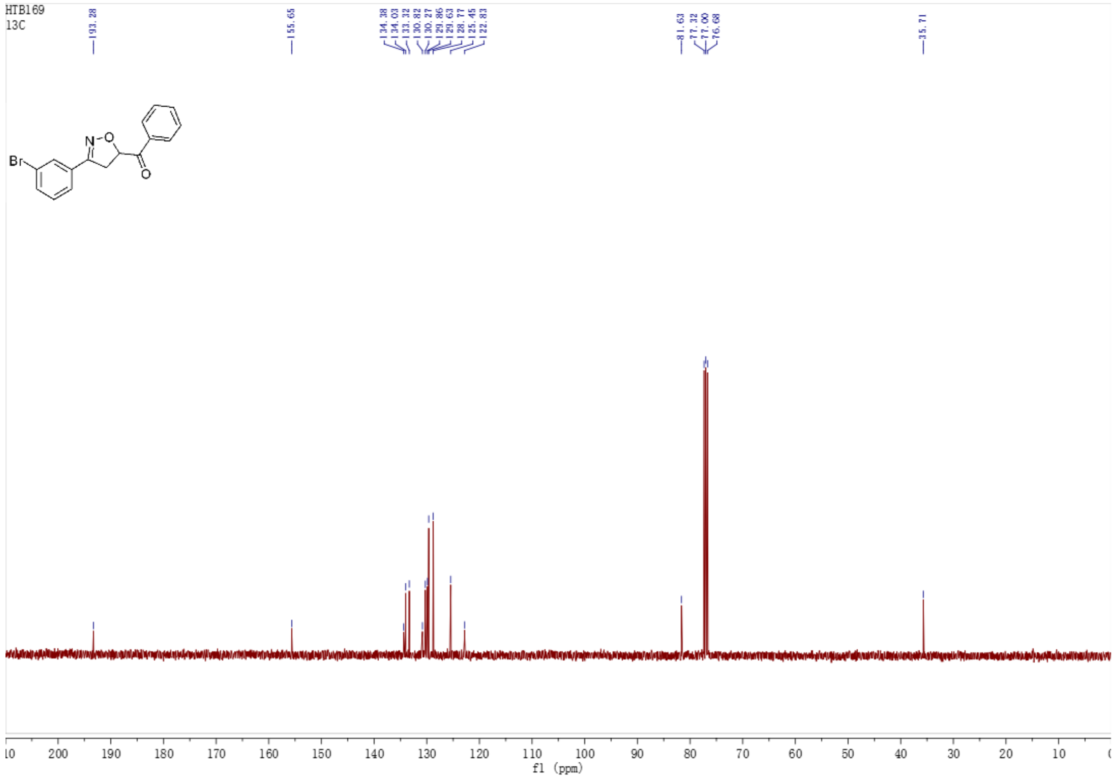

Figure S8. The ^{1}H NMR and ^{13}C NMR Spectrum of **3ha** in $CDCl_3$.

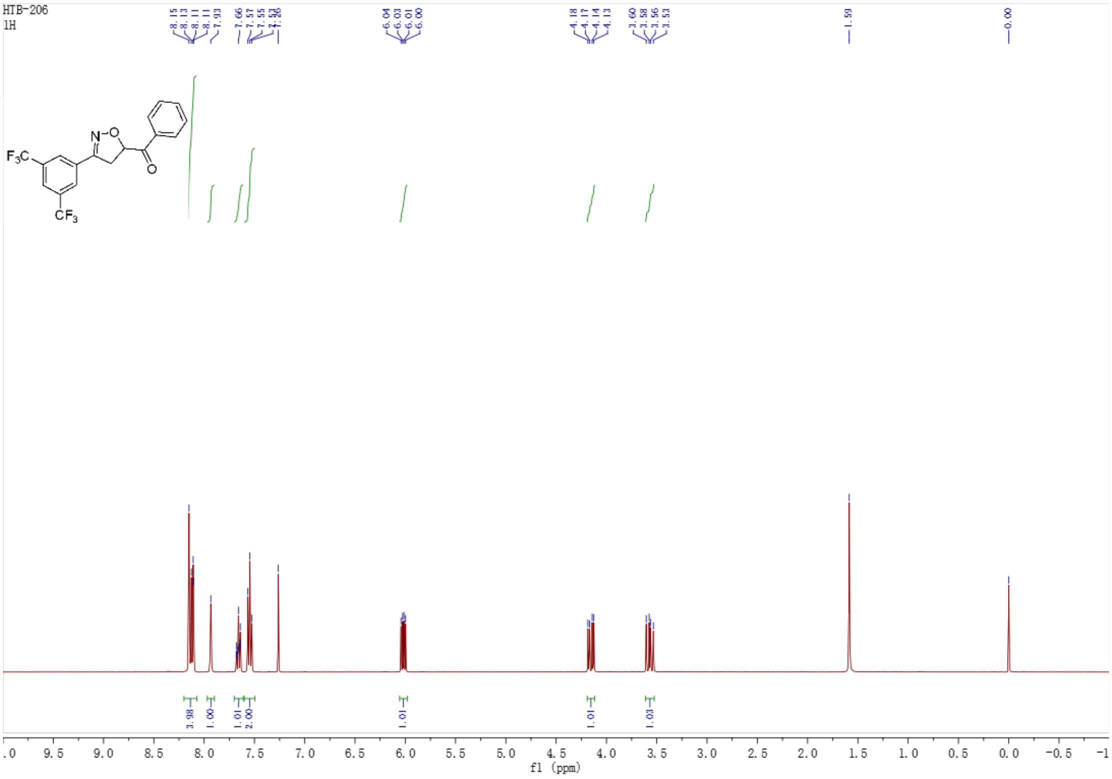

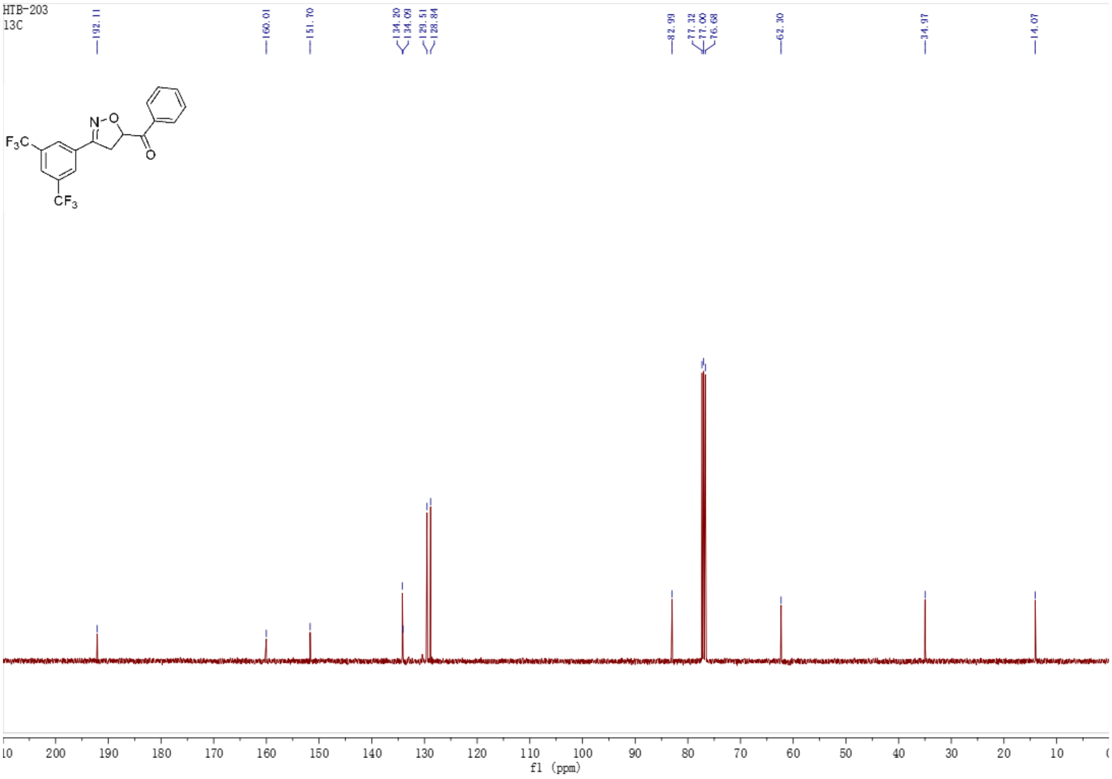

Figure S9-1. The ^{1}H NMR and ^{13}C NMR Spectrum of **3ia** in $CDCl_3$.

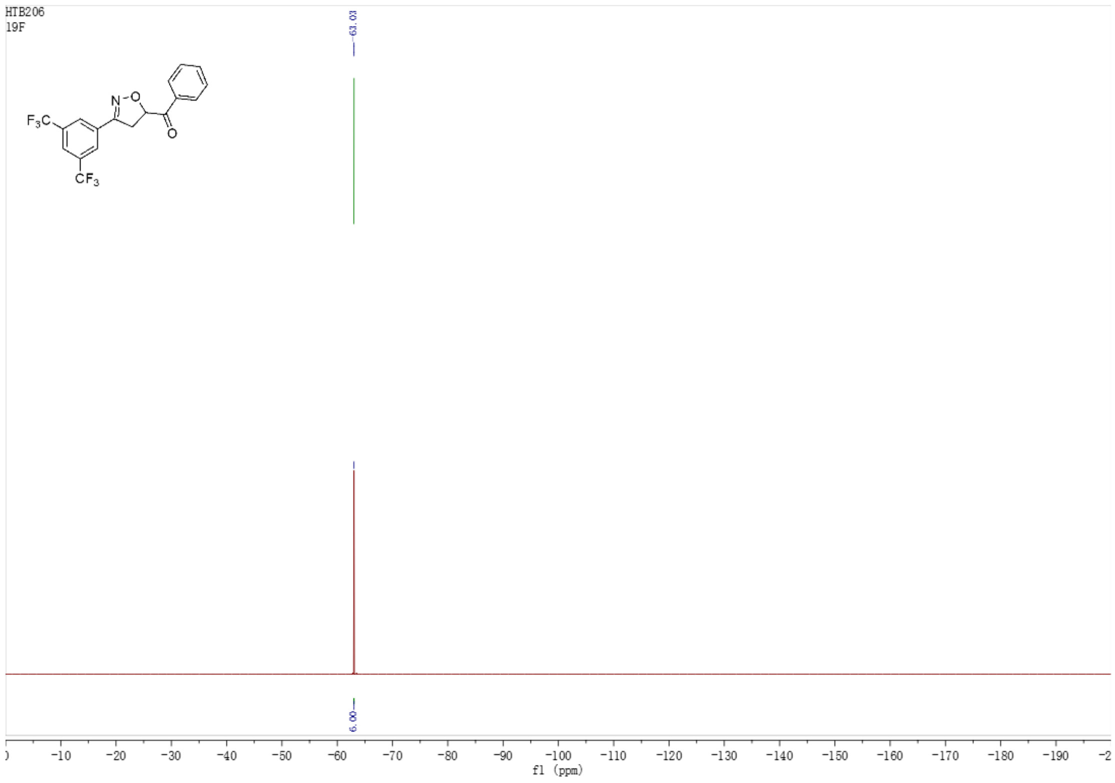

Figure S9-2. The ^{19}F NMR Spectrum of **3ia** in $CDCl_3$.

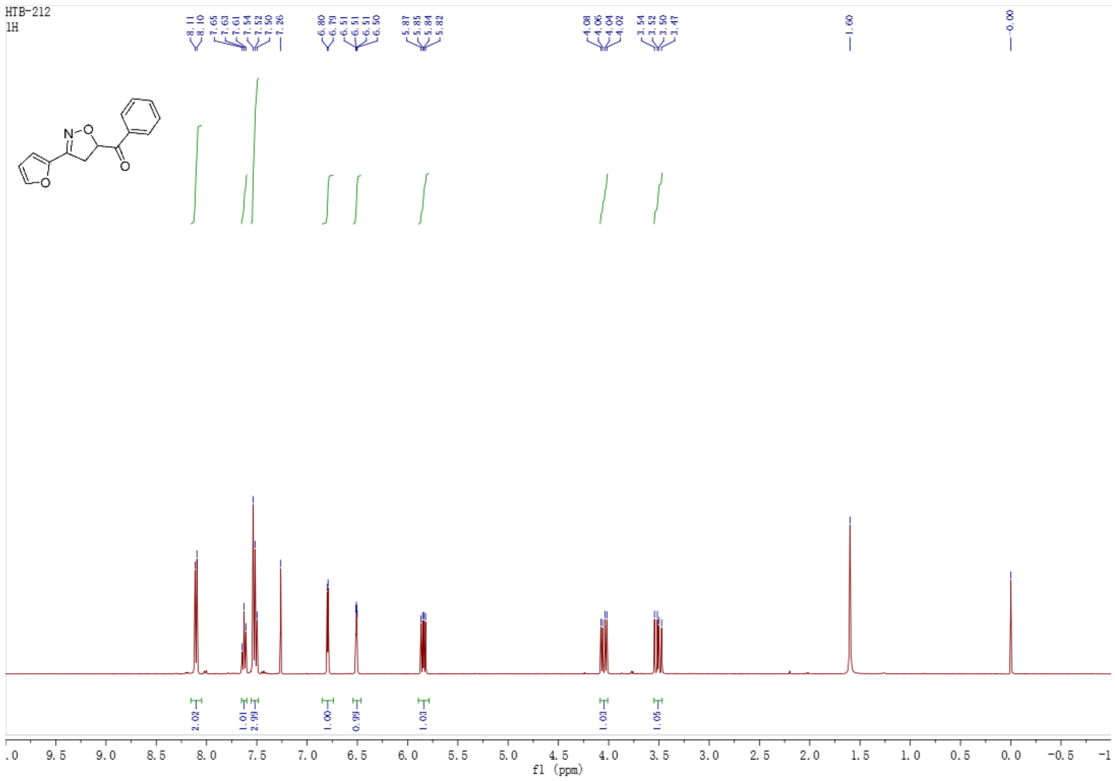

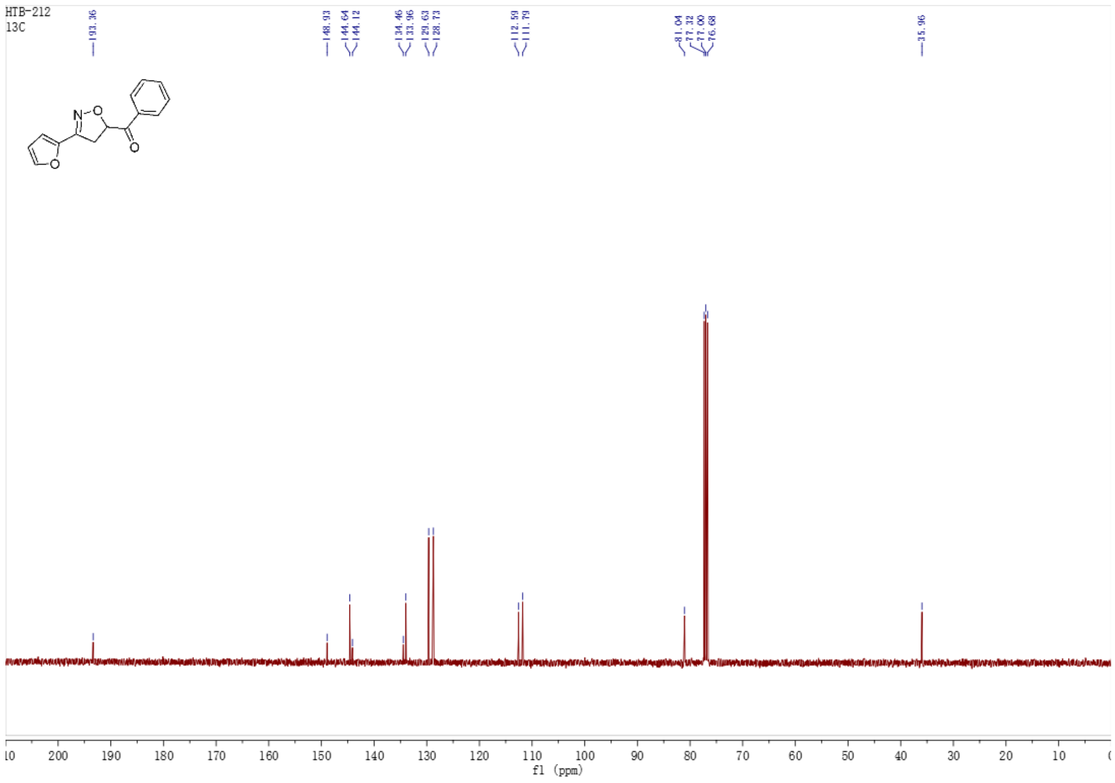

Figure S10. The ^{1}H NMR and ^{13}C NMR Spectrum of **3ja** in $CDCl_3$.

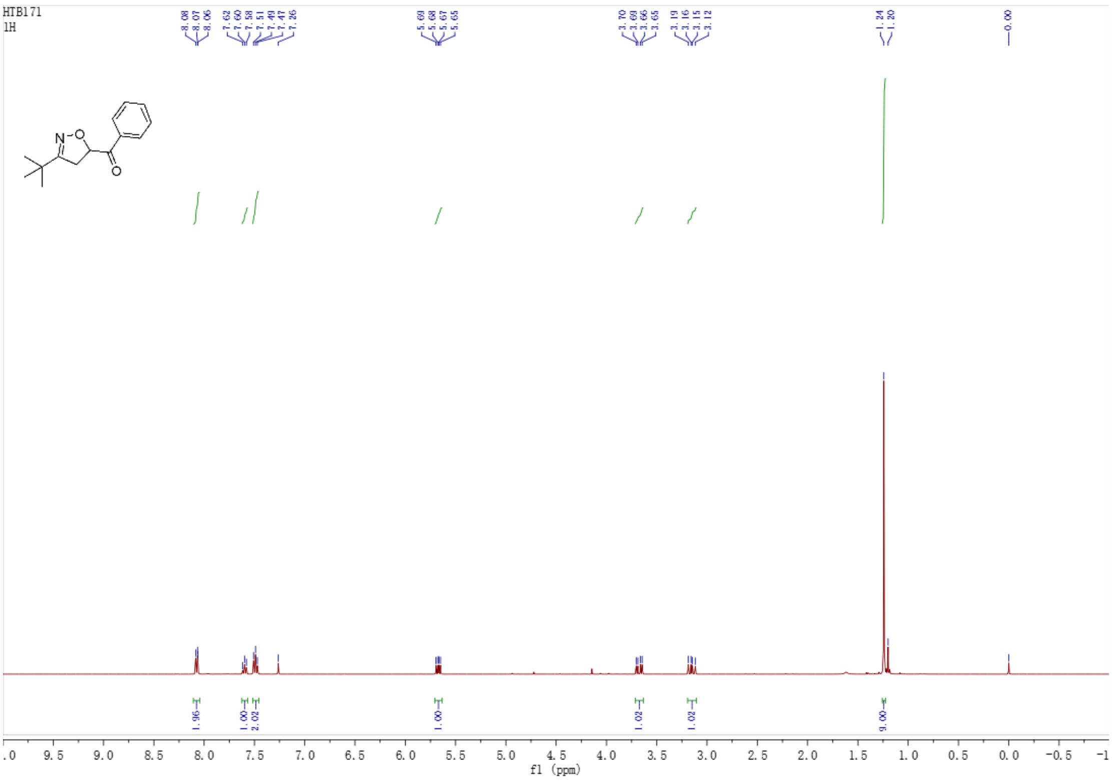

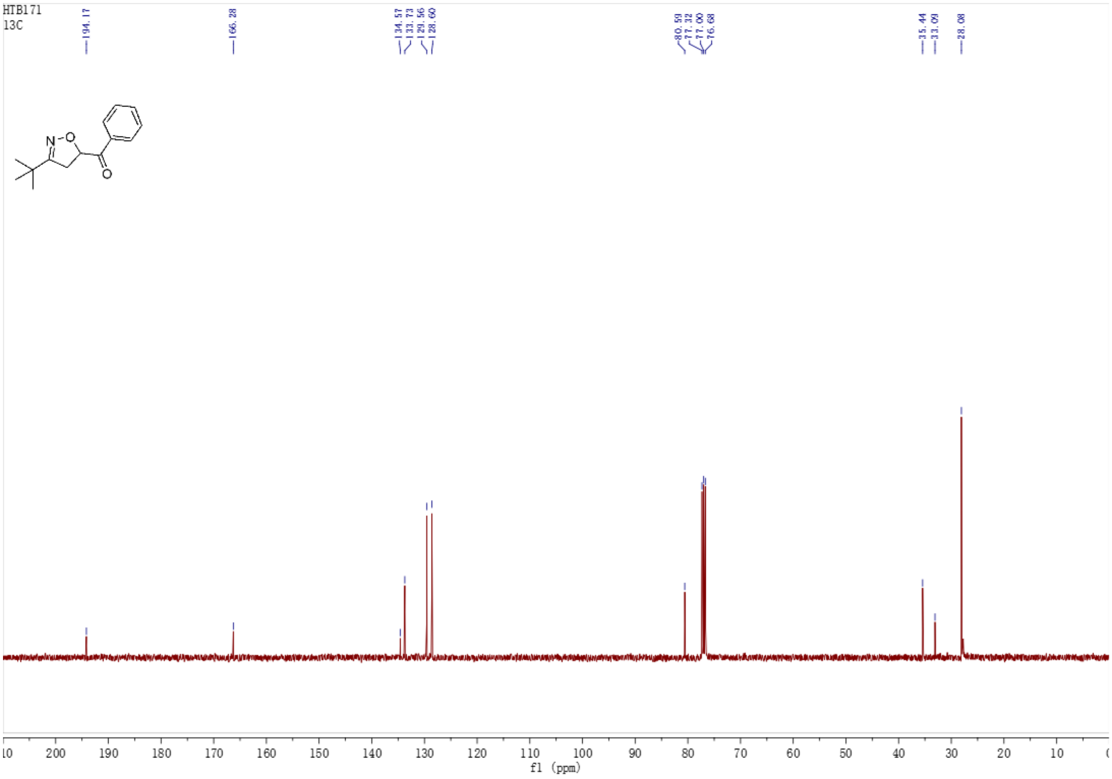

Figure S11. The ^{1}H NMR and ^{13}C NMR Spectrum of **3ka** in $CDCl_3$.

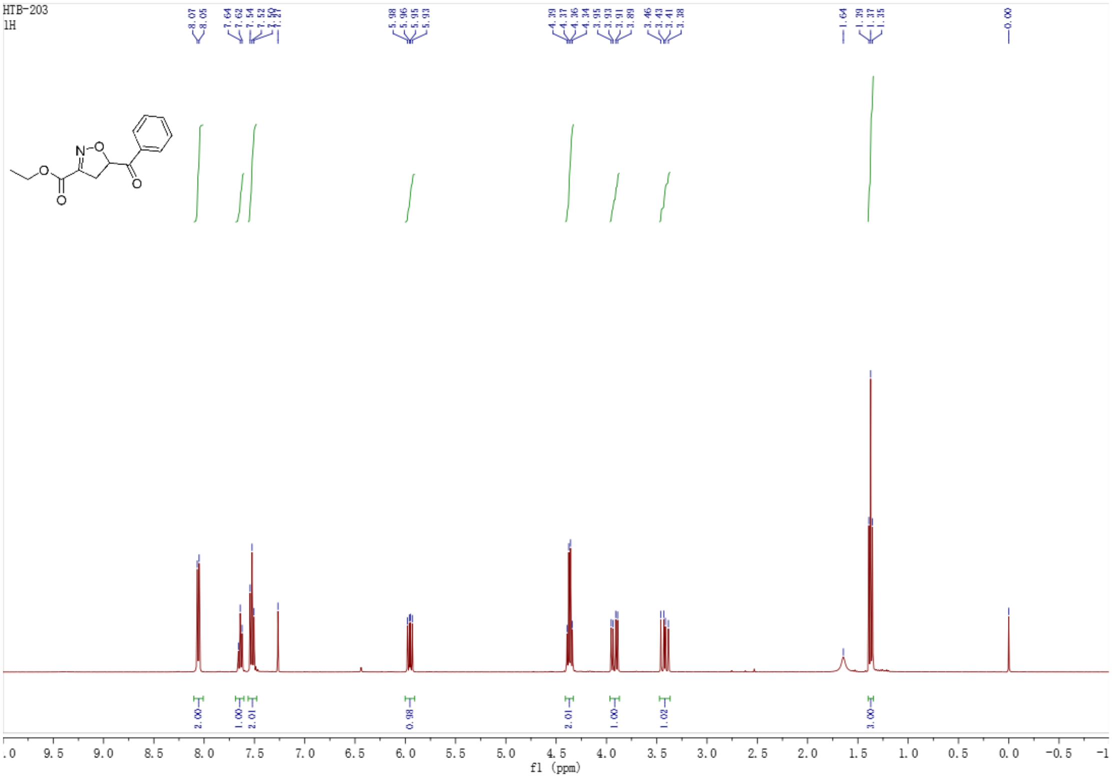

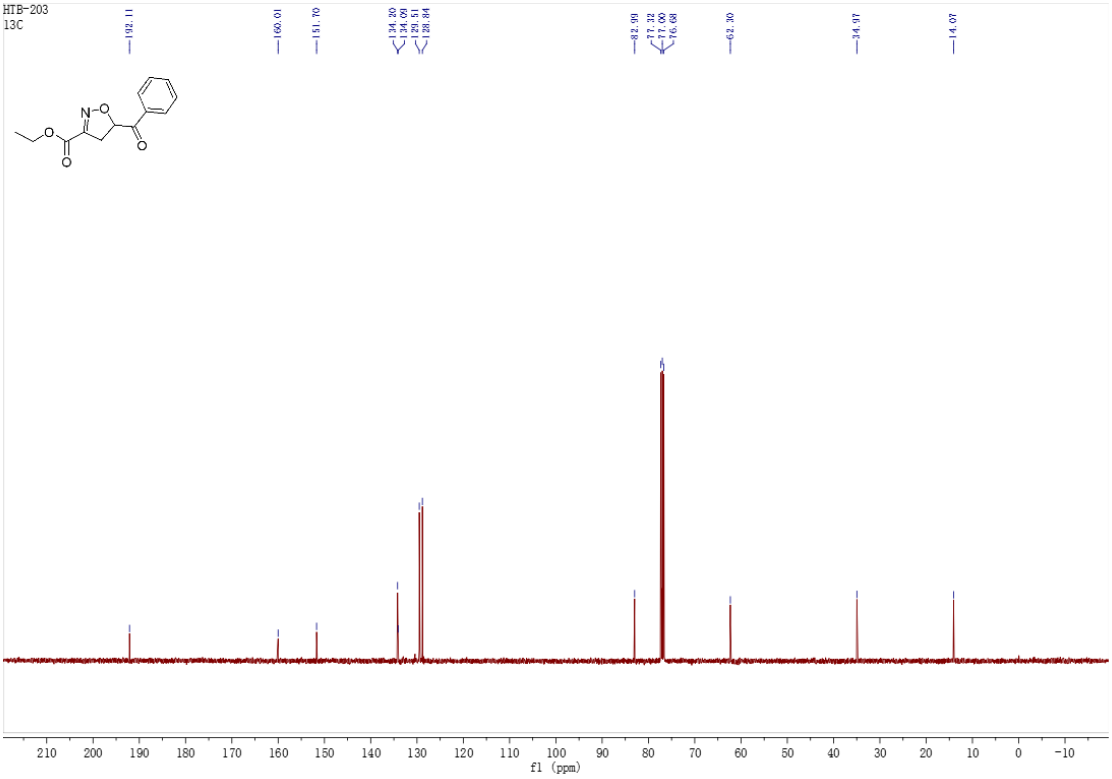

Figure S12. The ^{1}H NMR and ^{13}C NMR Spectrum of **3la** in $CDCl_3$.

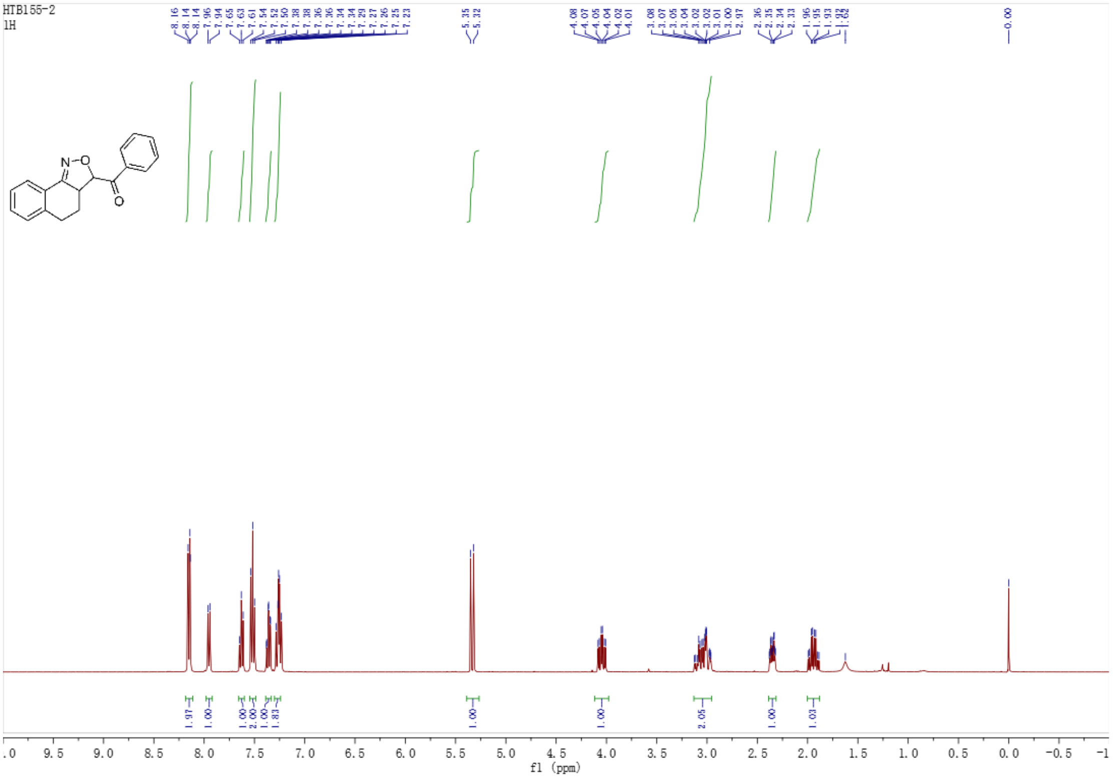

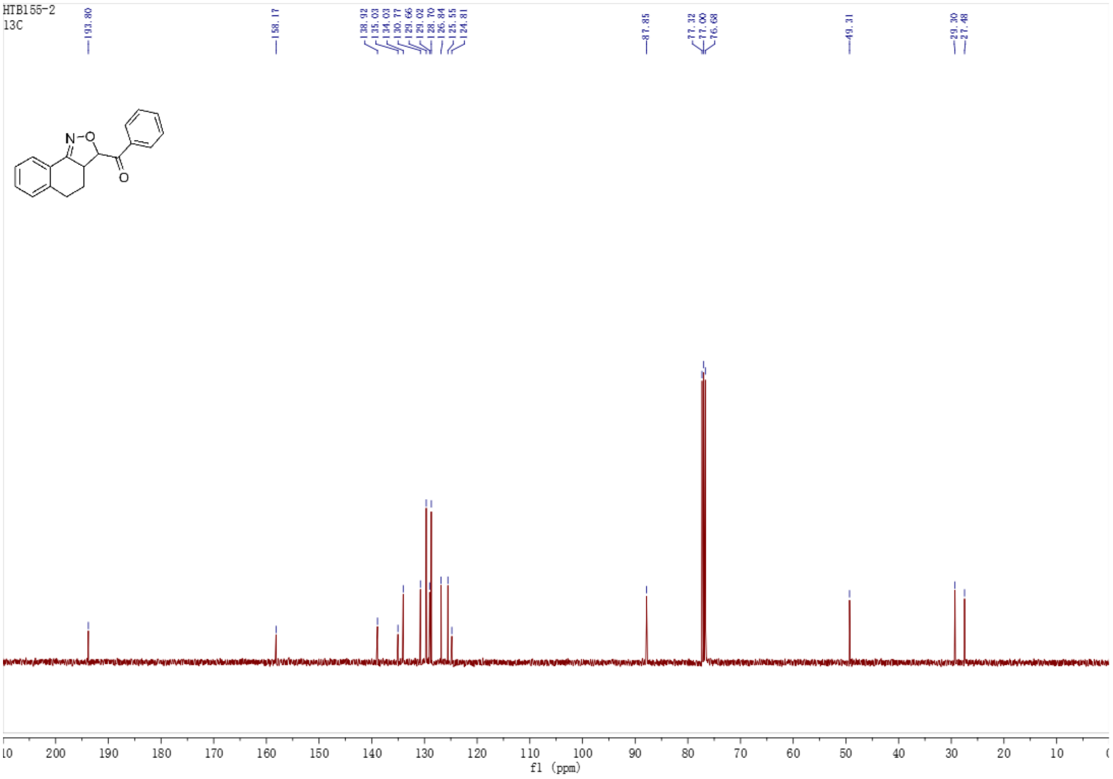

Figure S13. The ^{1}H NMR and ^{13}C NMR Spectrum of **3ma** in $CDCl_3$.

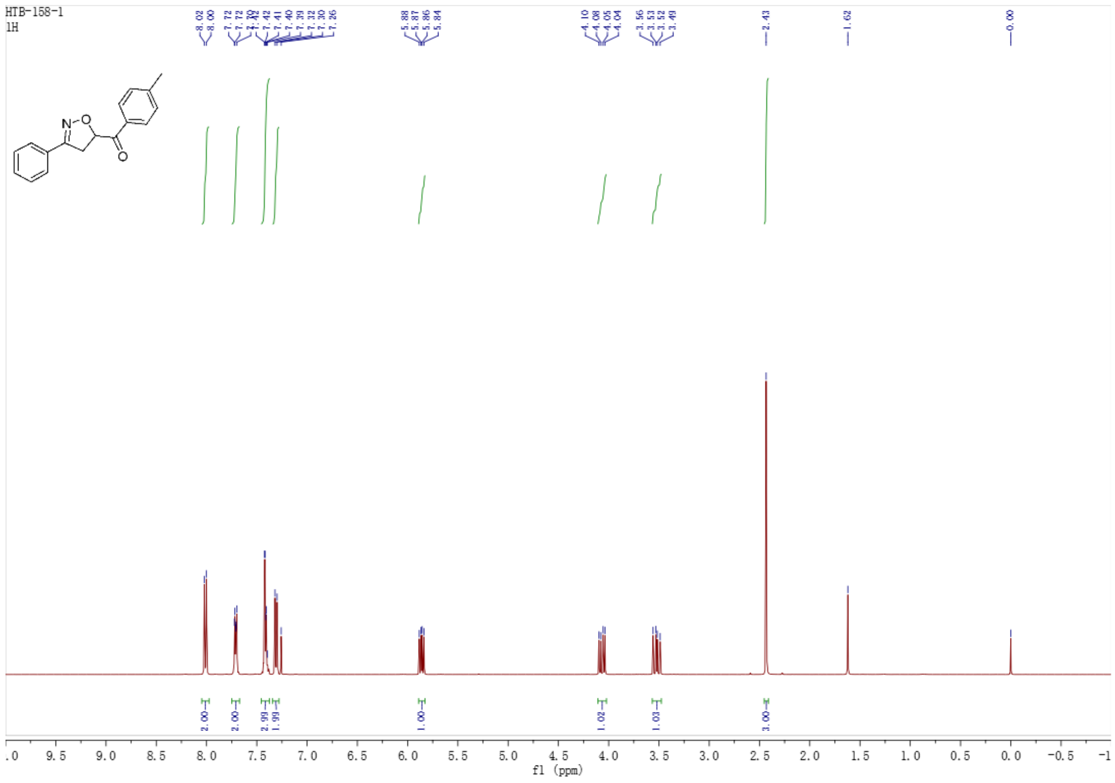

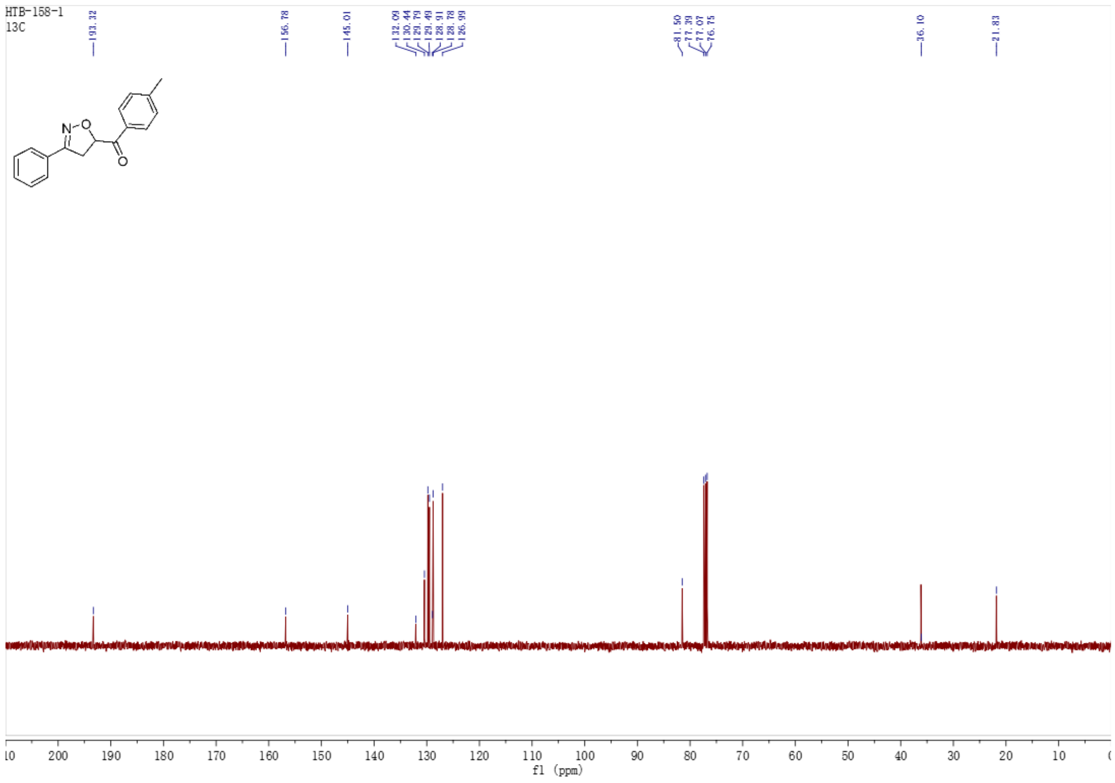

Figure S14. The ^{1}H NMR and ^{13}C NMR Spectrum of **3ab** in $CDCl_3$.

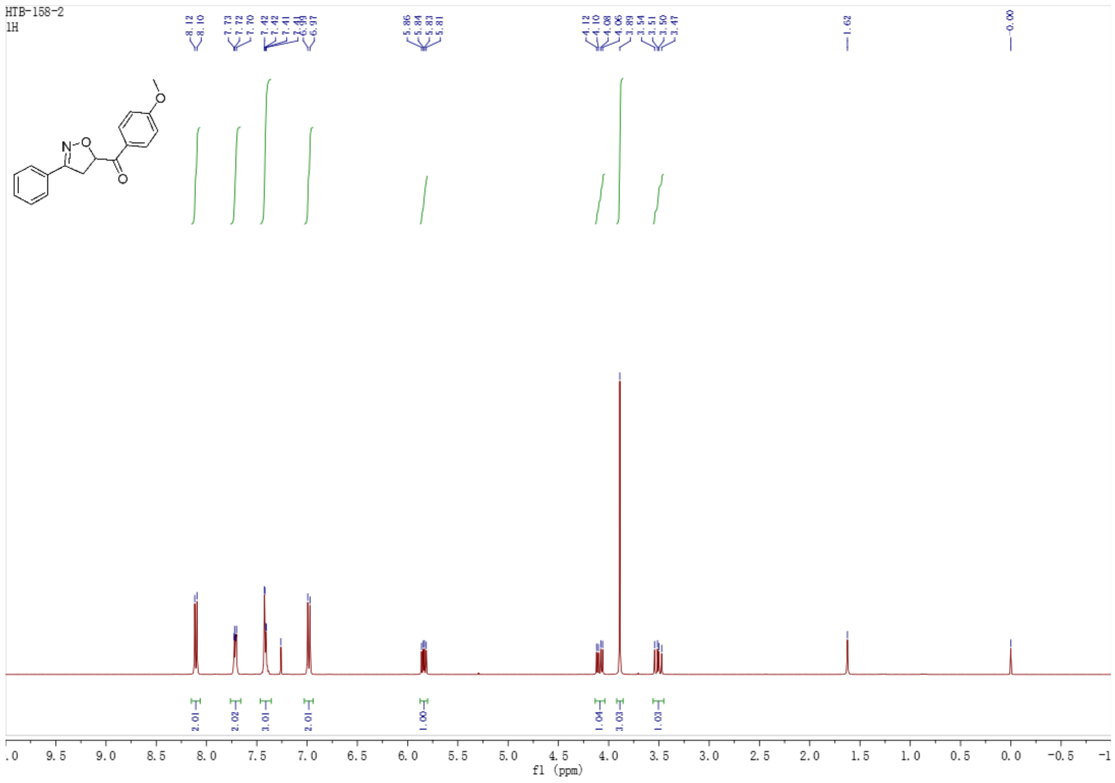

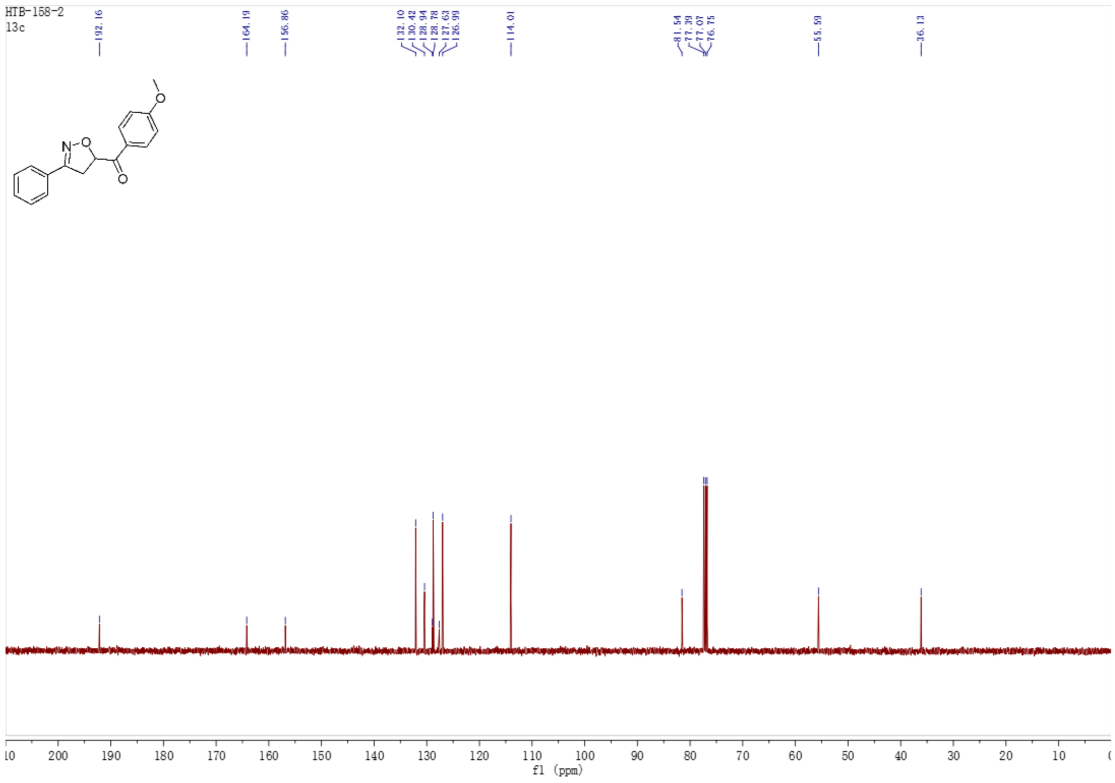

Figure S15. The ^{1}H NMR and ^{13}C NMR Spectrum of **3ac** in $CDCl_3$.

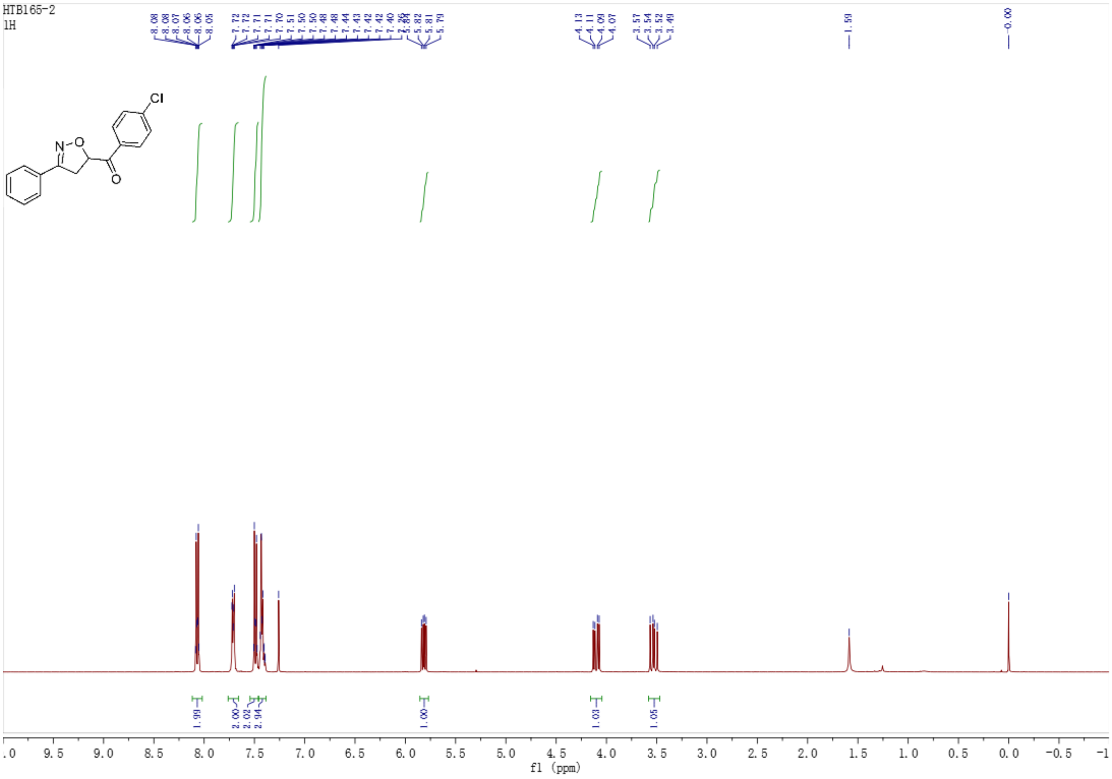

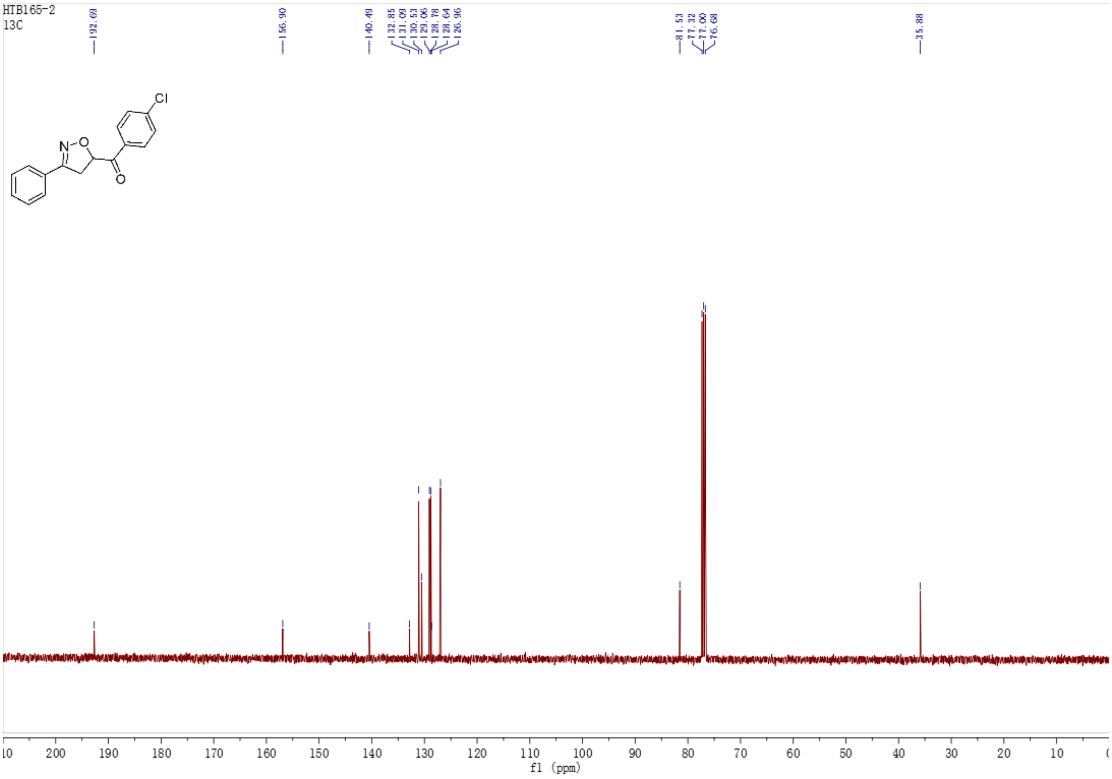

Figure S16. The ^{1}H NMR and ^{13}C NMR Spectrum of **3ad** in $CDCl_3$.

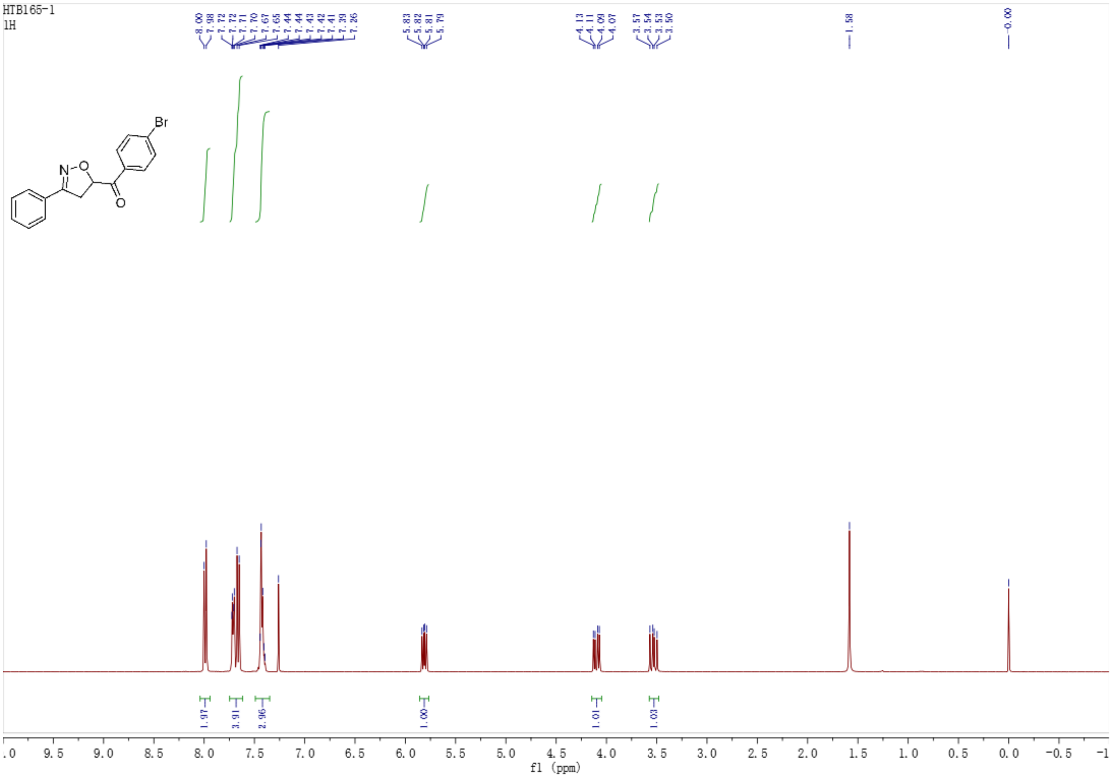

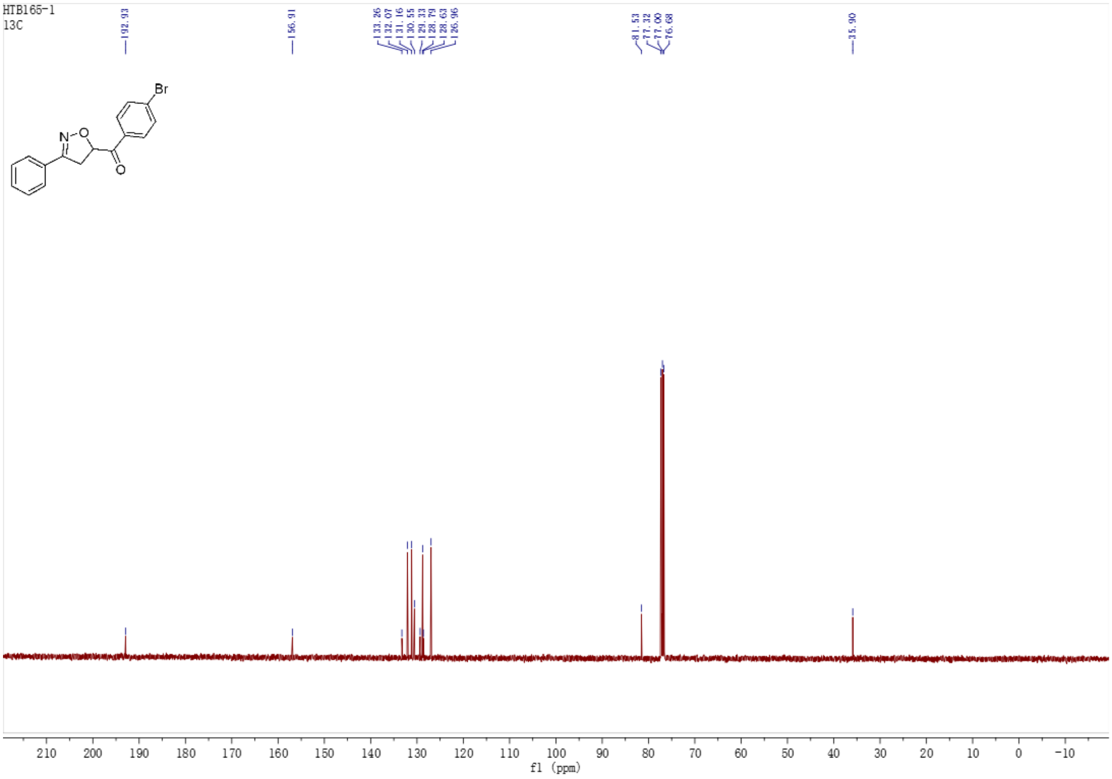

Figure S17. The ^{1}H NMR and ^{13}C NMR Spectrum of **3ae** in $CDCl_3$.

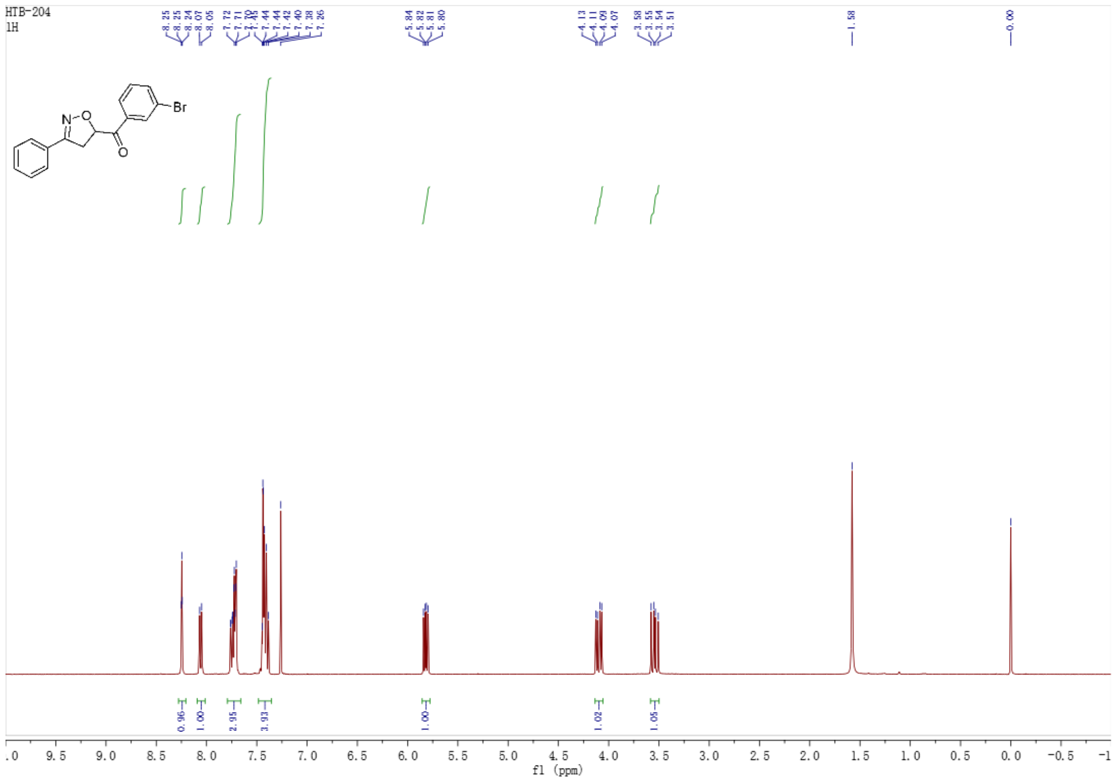

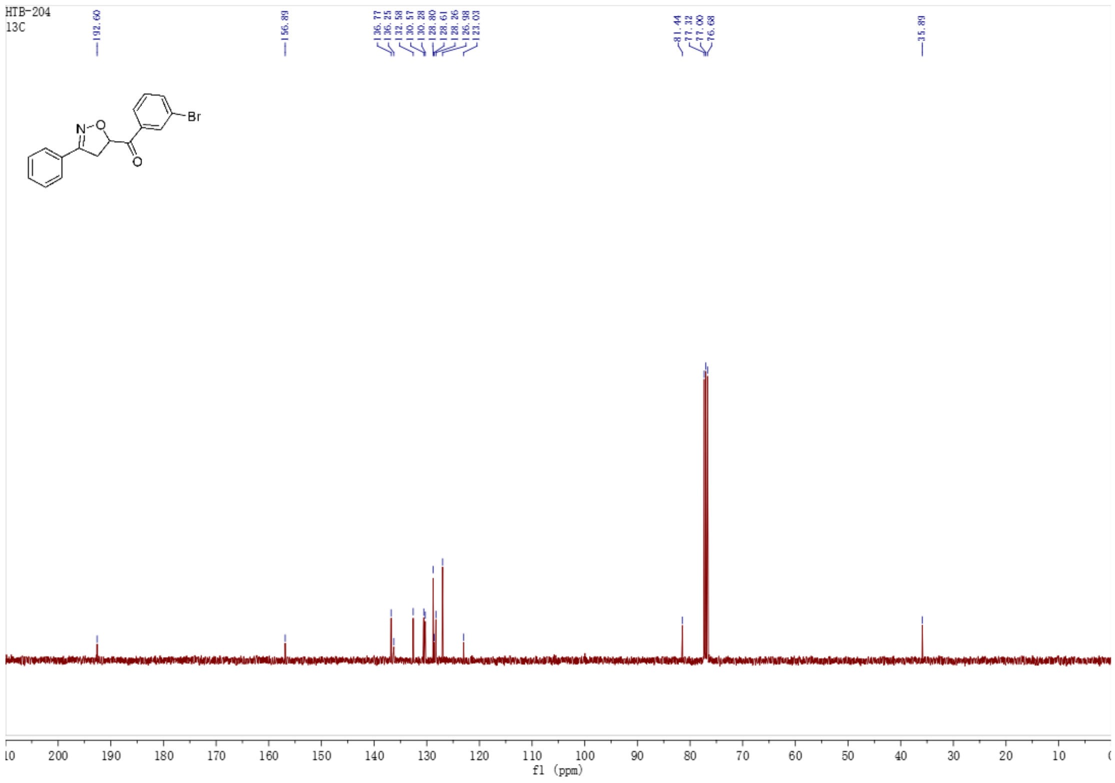

Figure S18. The ^{1}H NMR and ^{13}C NMR Spectrum of **3af** in $CDCl_3$.

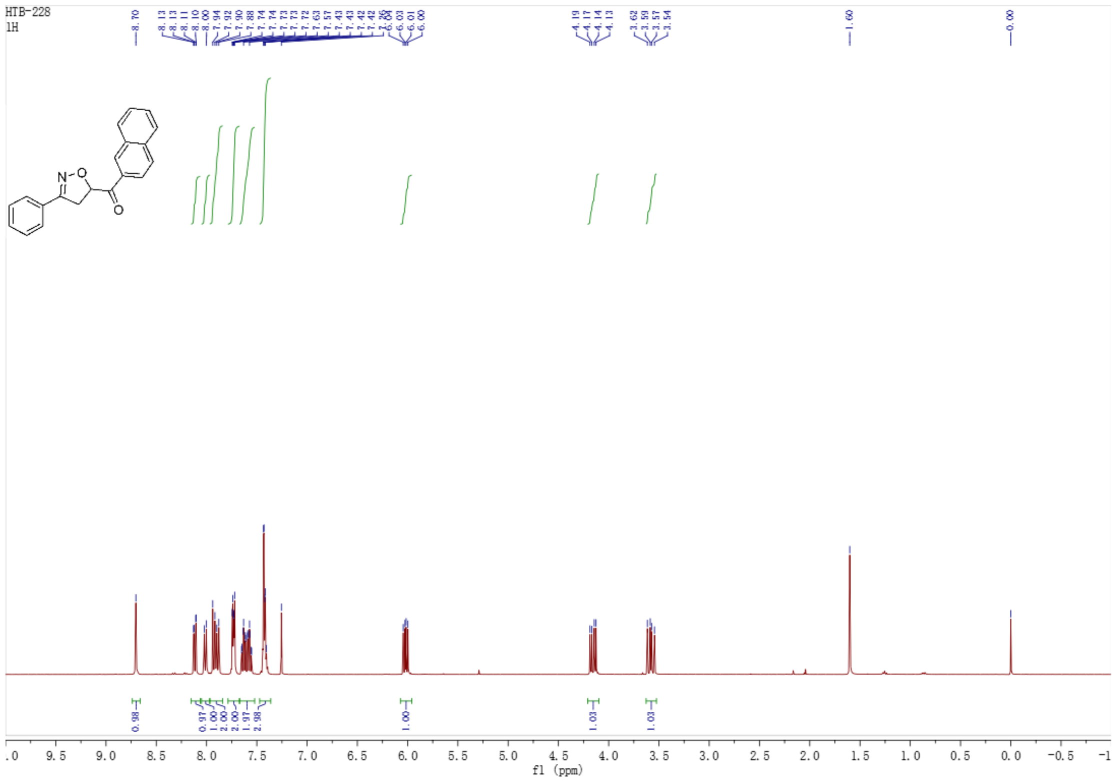

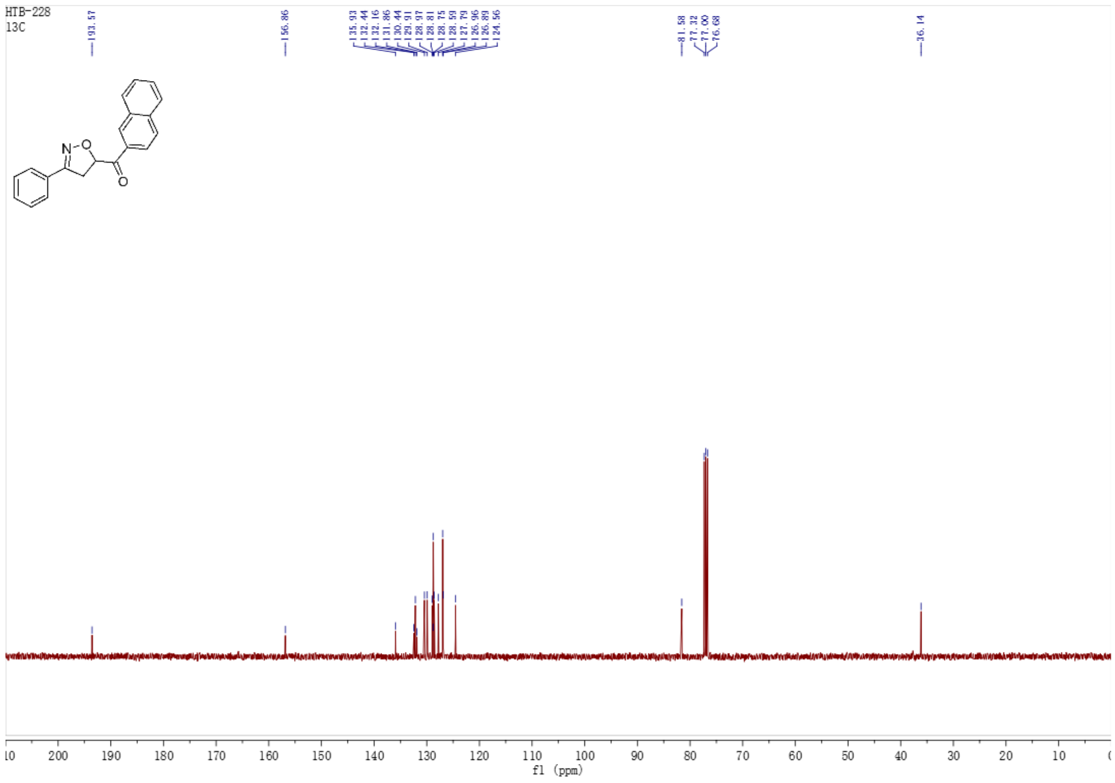

Figure S19. The ^{1}H NMR and ^{13}C NMR Spectrum of **3ag** in $CDCl_3$.

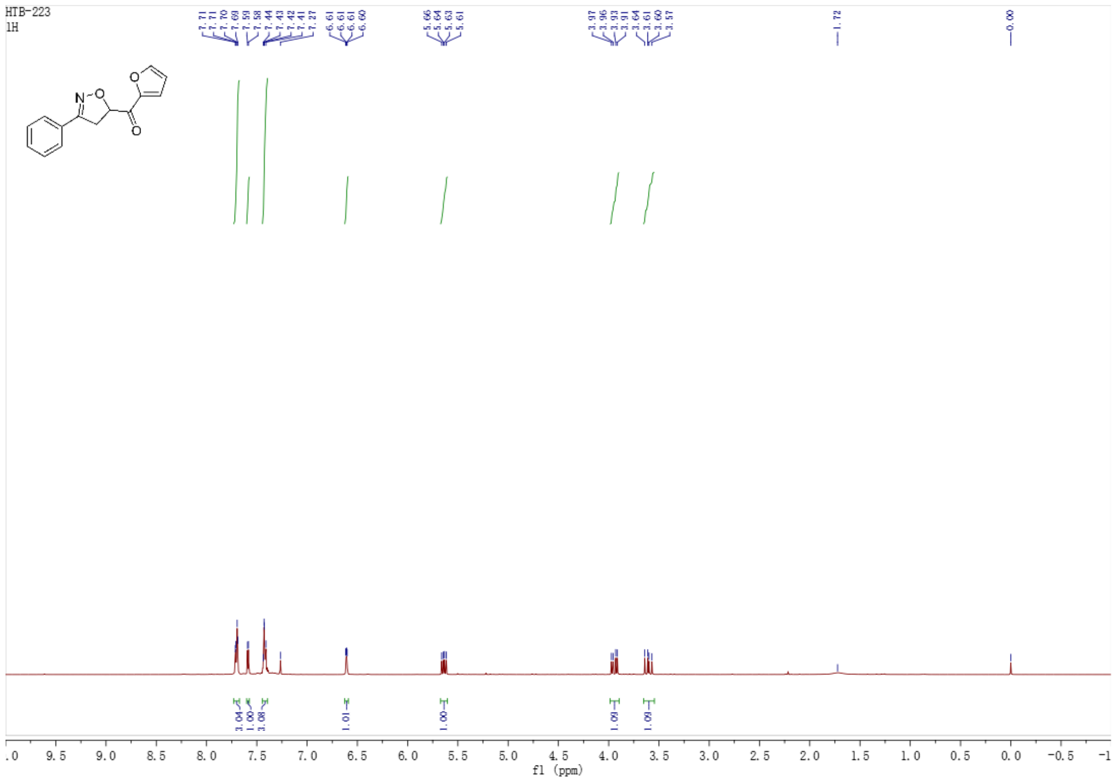

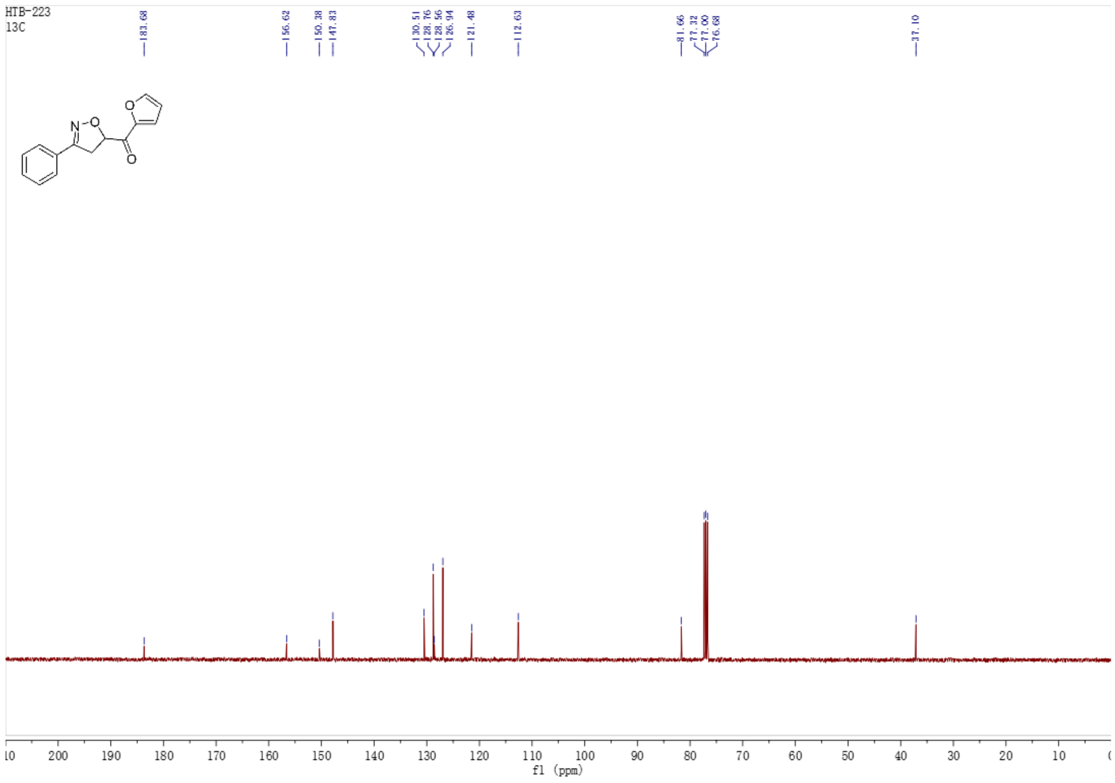

Figure S20. The ^{1}H NMR and ^{13}C NMR Spectrum of **3ah** in $CDCl_3$.

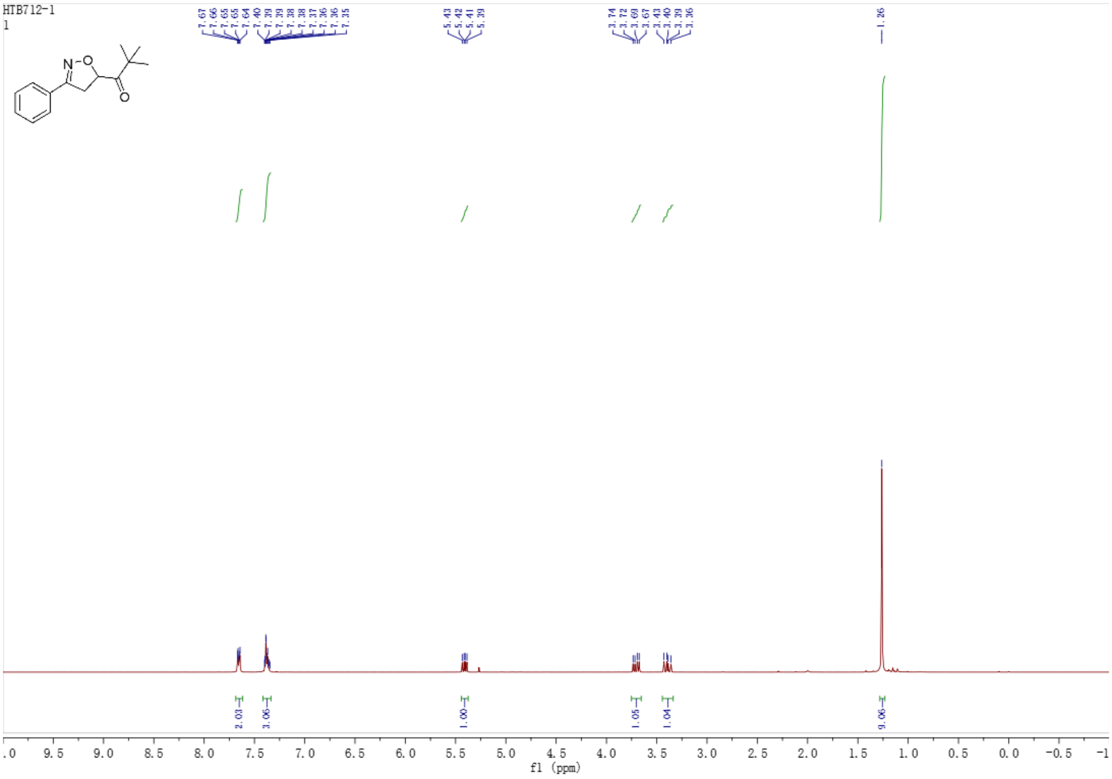

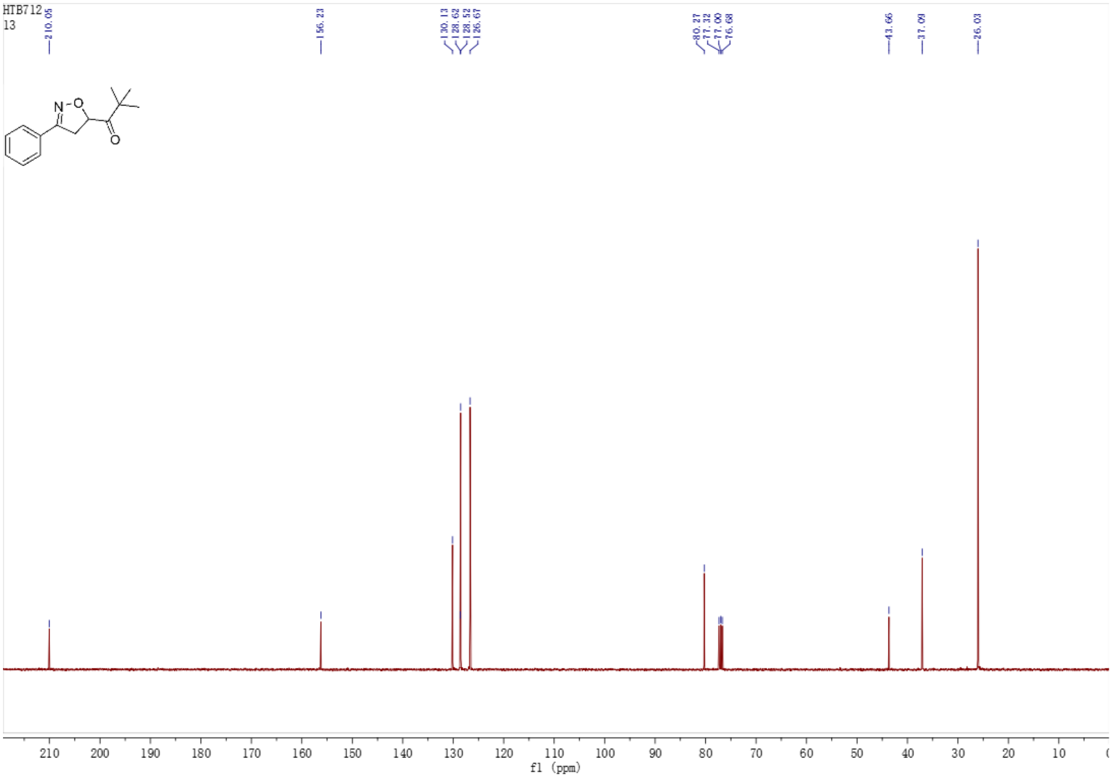

Figure S21. The ^{1}H NMR and ^{13}C NMR Spectrum of **3ai** in $CDCl_3$.

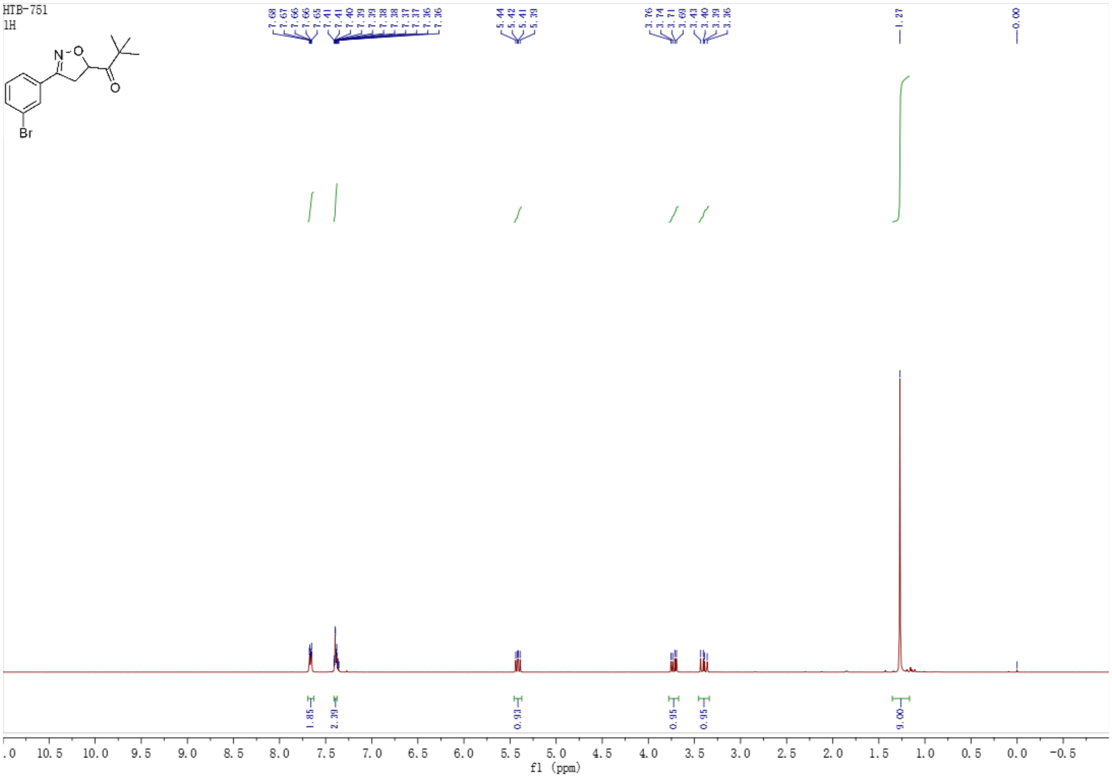

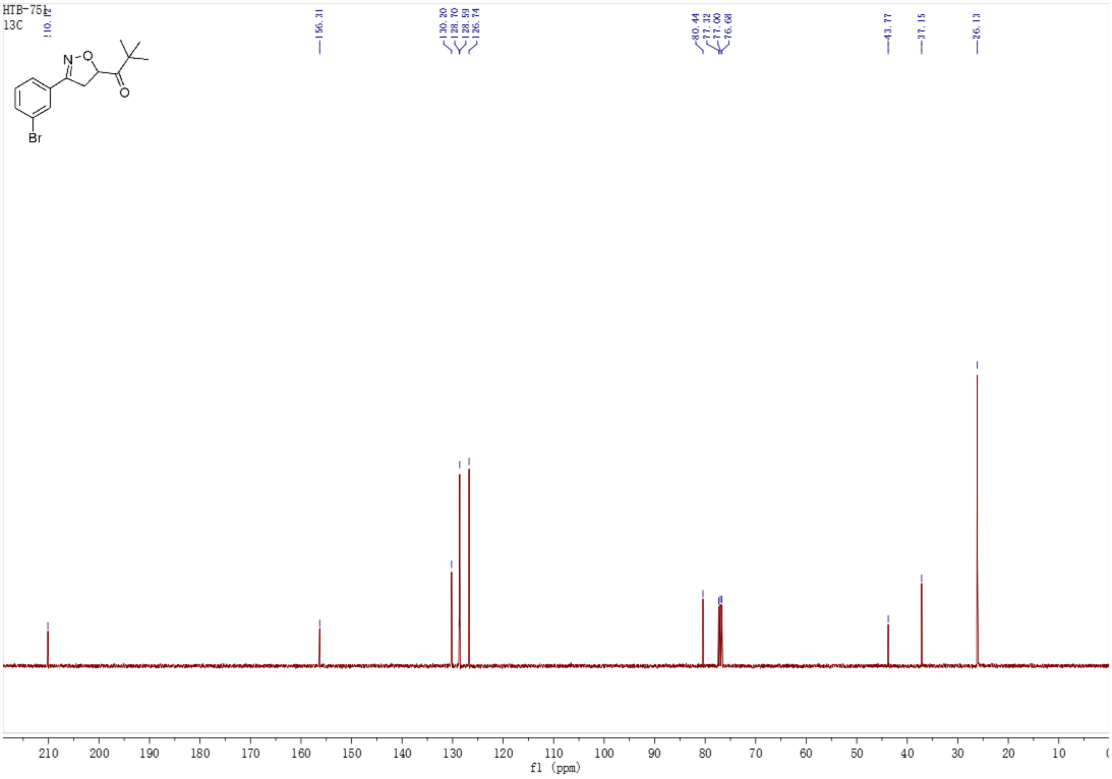

Figure S22. The ^{1}H NMR and ^{13}C NMR Spectrum of **3aj** in $CDCl_3$

7. ^{1}H NMR and ^{13}C NMR spectra of compounds 4aa.

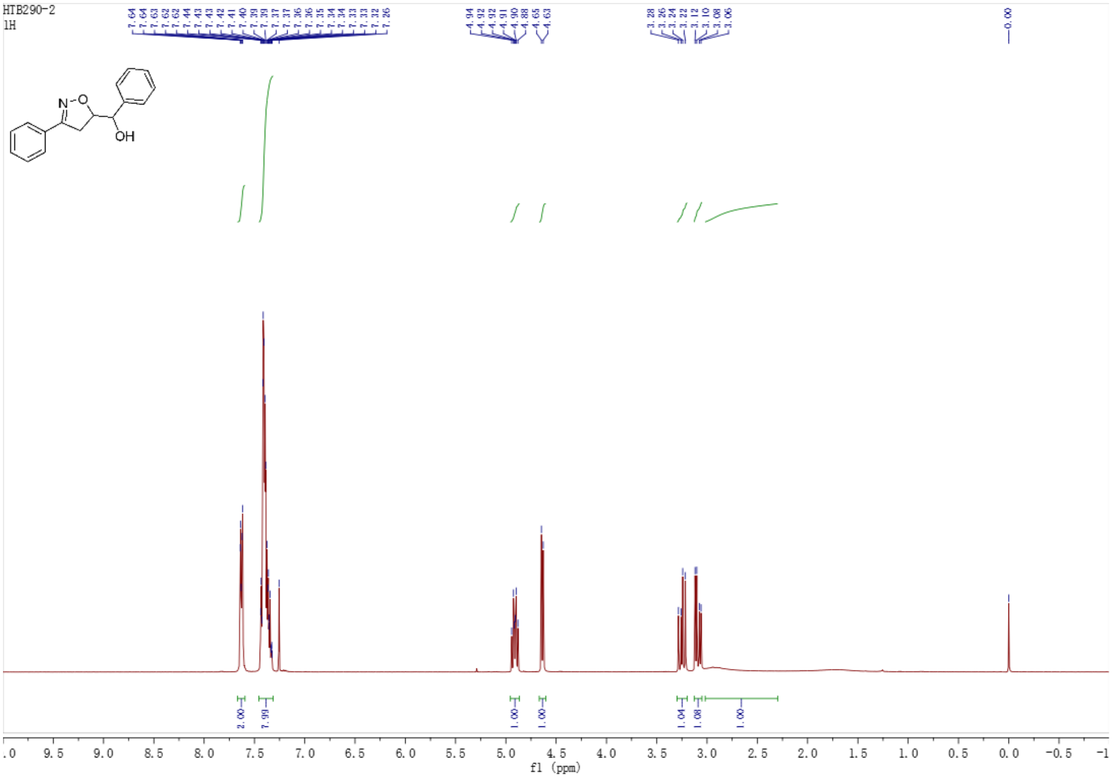

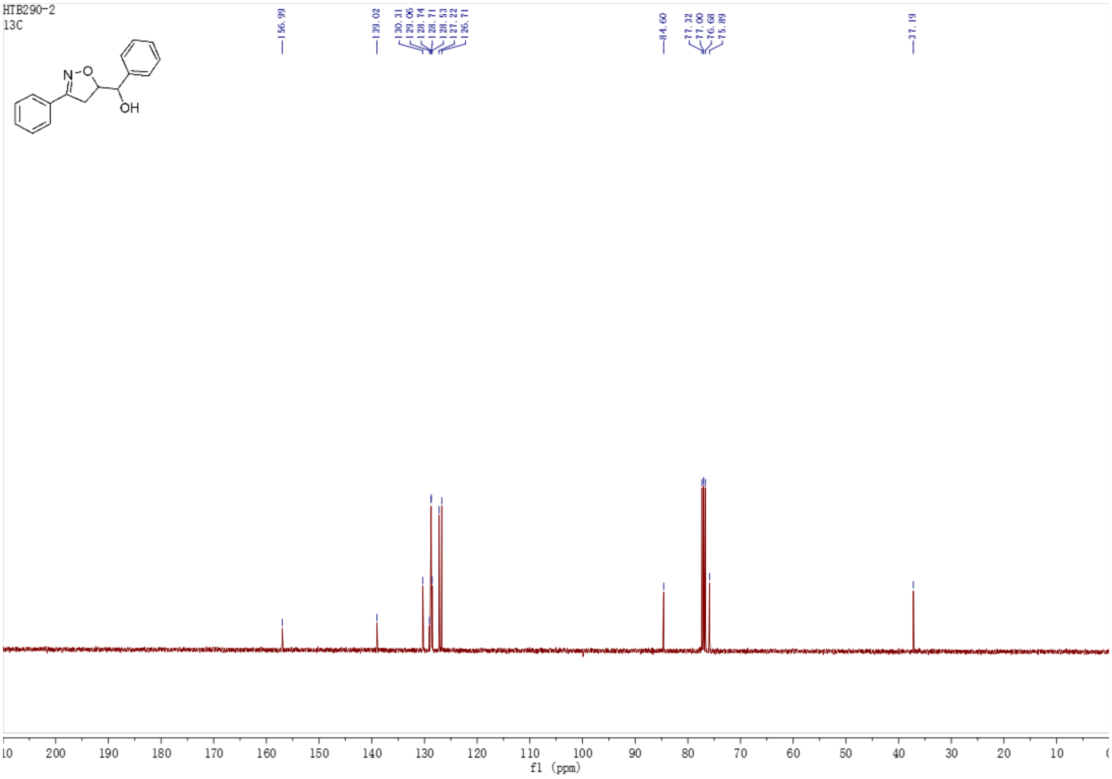

Figure S23. The ^{1}H NMR and ^{13}C NMR Spectrum of **4aa** in $CDCl_3$.

8. Copies of HPLC Chromatograms

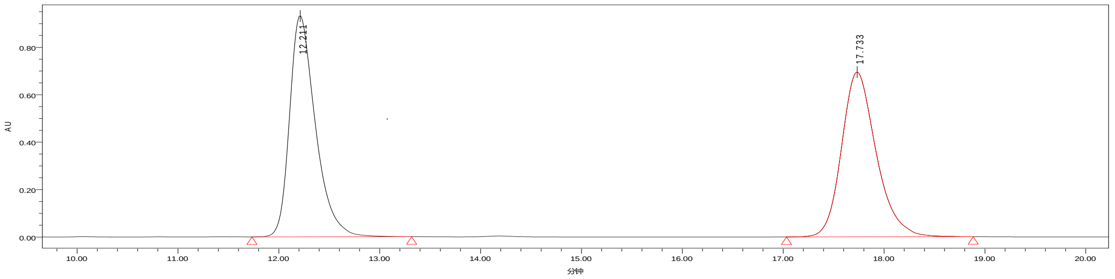

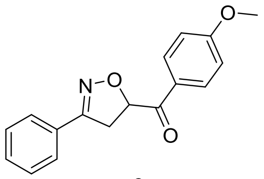

***rac*-3ac**

Peak	Ret. Time [min]	Type	Area [μAU·S]	Height [μAU]	Area %
**1**	12.211	BB	16601818	932554	50.08
**2**	17.733	BB	16551944	695044	49.92

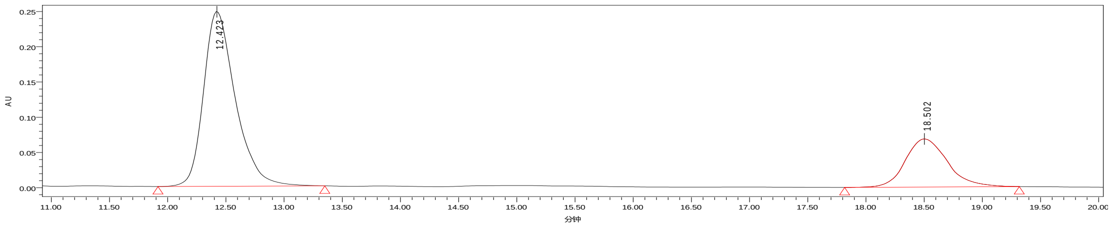

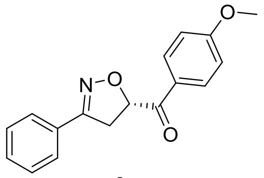

3ac

Peak	Ret. Time [min]	Type	Area [μAU·S]	Height [μAU]	Area %
**1**	12.423	BB	4707078	278181	73.37
**2**	18.502	BB	1708086	68270	26.63

Method: chiral IA-H, hexane/*i*PrOH = 70/30, 1 mL/min, 254 nm, 25 ^{o}C
